# Mitigating susceptibility-induced distortions in high-resolution 3DEPI fMRI at 7T

**DOI:** 10.1016/j.neuroimage.2023.120294

**Published:** 2023-10-01

**Authors:** Vahid Malekian, Nadine N Graedel, Alice Hickling, Ali Aghaeifar, Barbara Dymerska, Nadège Corbin, Oliver Josephs, Eleanor A. Maguire, Martina F. Callaghan

**Affiliations:** a Wellcome Centre for Human Neuroimaging, UCL Queen Square Institute of Neurology, University College London, UK; b MR Research Collaborations, Siemens Healthcare Limited, Frimley, UK; c Centre de Résonance Magnétique des Systèmes Biologiques, CNRS‐University Bordeaux, Bordeaux, France

**Keywords:** Distortion correction, fMRI, EPI, Ultra high field, High-resolution, Magnetisation transfer

## Abstract

Geometric distortion is a major limiting factor for spatial specificity in high-resolution fMRI using EPI readouts and is exacerbated at higher field strengths due to increased B_0_ field inhomogeneity. Prominent correction schemes are based on B_0_ field-mapping or acquiring reverse phase-encoded (reversed-PE) data. However, to date, comparisons of these techniques in the context of fMRI have only been performed on 2DEPI data, either at lower field or lower resolution. In this study, we investigate distortion compensation in the context of sub-millimetre 3DEPI data at 7T. B_0_ field-mapping and reversed-PE distortion correction techniques were applied to both partial coverage BOLD-weighted and whole brain MT-weighted 3DEPI data with matched distortion. Qualitative assessment showed overall improvement in cortical alignment for both correction techniques in both 3DEPI fMRI and whole-brain MT-3DEPI datasets. The distortion-corrected MT-3DEPI images were quantitatively evaluated by comparing cortical alignment with an anatomical reference using dice coefficient (DC) and correlation ratio (CR) measures. These showed that B_0_ field-mapping and reversed-PE methods both improved correspondence between the MT-3DEPI and anatomical data, with more substantial improvements consistently obtained using the reversed-PE approach. Regional analyses demonstrated that the largest benefit of distortion correction, and in particular of the reversed-PE approach, occurred in frontal and temporal regions where susceptibility-induced distortions are known to be greatest, but had not led to complete signal dropout. In conclusion, distortion correction based on reversed-PE data has shown the greater capacity for achieving faithful alignment with anatomical data in the context of high-resolution fMRI at 7T using 3DEPI.

## Highlights

• Performance of two distortion correction methods was assessed for laminar fMRI at 7T.
• B0 field-mapping and reversed phase encoding methods reduced distortion in 3DEPI data.
• Greater improvements were consistently obtained using the reversed phase encoding method.
• Magnetisation transfer weighting improves contrast and eases cortical segmentation.

## 1 Introduction

In human neuroscience, there is increasing demand to improve our understanding of neuronal processing in the layers of the cortex using functional MRI (Kashyap et al., 2018, Lawrence et al., 2019, Self et al., 2019). This has driven technical developments in hardware, acquisition strategies and post-processing (Wald, 2012, Polimeni et al., 2018, Malekian et al., 2020). For example, ultra-high field (UHF) (≥7T) imaging increases the signal to noise ratio (SNR), which can be traded for higher spatial resolution, and comes with a concomitant boost in spatial specificity of the BOLD signal by suppressing its intravascular component (Yacoub et al., 2007, Uludag et al., 2009, Malekian et al., 2018). *In vivo* fMRI at 7T is now capable of dissociating signals originating from deep, middle and superficial cortical layers (Dumoulin et al., 2018, Muckli et al., 2015, Kok et al., 2016, Aitken et al., 2020, Iamshchinina et al., 2020, Ng et al., 2021, van Dijk et al., 2021), i.e. "depth-resolved fMRI" or “laminar fMRI”.

However, spatial specificity can be compromised by inhomogeneity in the static magnetic field, B_0_. This inhomogeneity, originating from susceptibility differences within the head, scales with the strength of B_0_ and is therefore particularly problematic at UHF. When using the echo-planar imaging (EPI) technique, crucial to functional MRI because of its rapidity, this susceptibility-induced effect leads to image distortions along the low bandwidth phase-encoded direction. Several methods have been suggested to mitigate geometric distortion in EPI data. For example, the data acquisition step can be altered by using advanced shimming techniques to improve B_0_ homogeneity (Volz et al., 2019, Stockmann and Wald, 2018) or by shortening the readout using one or a combination of segmentation (Stirnberg and Stocker, 2020), parallel imaging (Moeller et al., 2006), and increasing the bandwidth to reduce the echo spacing. However, each solution has its own drawbacks such as lengthening the volume acquisition time (segmentation), or reducing SNR (parallel imaging, increasing bandwidth). Instead, or in addition to, altering the acquisition, distortion can be corrected in post-processing by integrating knowledge about the B_0_ field inhomogeneity. This is most commonly done by directly measuring the B_0_ field (Hutton et al., 2002, Zeng and Constable, 2002, Jezzard and Balaban, 1995, Matakos et al., 2014) or by acquiring images with reversed phase-encoding direction (reversed-PE) (Andersson et al., 2003, Morgan et al., 2004, Holland et al., 2010, Hedouin et al., 2017) and subsequently estimating the field that has produced these data in a model-based approach (Chang and Fitzpatrick, 1992), e.g. using the “topup” algorithm implemented in the FMRIB Software Library (FSL) (Andersson et al., 2003). The estimated field maps, obtained by either of the approaches, are then applied to the actual EPI data in a final step to correct distortion.

Previous studies have reported improved performance for the reversed-PE approach relative to correction with measured B_0_ field-maps in the context of diffusion weighted imaging (DWI) (Esteban et al., 2014, Graham et al., 2017, Ruthotto et al., 2013) and fMRI (Holland et al., 2010, Fritz et al., 2014, Schallmo et al., 2021, Hong et al., 2015, Yamamoto et al., 2021), including at UHF (Fritz et al., 2014, Schallmo et al., 2021, Hong et al., 2015, Yamamoto et al., 2021). However significant differences in study design hinder direct translation of these results to high-resolution fMRI applications at UHF. The investigations in the context of DWI have used spin-echo images for the reversed-PE correction, which do not suffer from susceptibility-induced signal losses that degrade the quality of gradient-echo (GE) EPI. The fMRI studies have used two dimensional (2D) GE-EPI with moderate resolution (e.g. 1.2 mm (Schallmo et al., 2021) and 1.6 mm isotropic (Yamamoto et al., 2021)).

To reach the finer sub-millimetre spatial resolution required for laminar studies, 3D GE-EPI may be preferred to avoid imperfect slice profile effects (Muftuler and Nalcioglu, 2000, Poser et al., 2010, van der Zwaag et al., 2012) and to gain SNR from volumetric sampling in the thermal-noise-dominated regime of small voxels (van der Zwaag et al., 2012). However, BOLD-weighted 3DEPI suffers from reduced image contrast relative to 2DEPI if the flip angle is optimised to maximise grey matter signal. This can be problematic for post-processing steps that require accurate registration, alignment and cortical segmentation. Preparation modules can be used to boost contrast by introducing T_1_ (Kashyap et al., 2018, Ikonomidou et al., 2005, Renvall et al., 2016, Chai et al., 2021, Huber et al., 2017, van der Zwaag et al., 2018) or magnetisation transfer (MT) weighting (Chai et al., 2021, Schulz et al., 2020).

In this study, we aimed to mitigate distortions with two distortion correction techniques and assess their relative merit in high resolution GE BOLD-weighted fMRI by: (i) utilising 3DEPI in order to facilitate high spatial resolution in conjunction with high acceleration factors; (ii) employing a segmented readout to reduce susceptibility-induced distortions; (iii) augmenting the acquisition to integrate reversed-PE EPI volumes at the outset of each functional run prior to commencing the cognitive paradigm; (iv) implementing a preparatory module to impart MT-weighting, enabling the acquisition of higher contrast images with a matched readout in the same session. B_0_ field-mapping data were additionally acquired to assess the relative performance of the integrated reversed-PE approach for distortion correction. This was quantified for both distortion-correction of the EPI data in structural space (Marques et al., 2010) and distortion of “anatomically-faithful” MP2RAGE data in the EPI spaces. Dice coefficient and correlation ratio metrics were used to quantitatively augment qualitative visual assessment of the distortion correction performance of each method. To explore regional variability in performance, the assessment was performed by parcellating the cortex using the Harvard-Oxford cortical atlas (Desikan et al., 2006).

## 2 Methods

### 2.1 Sequence development

To address the issue of low tissue contrast in 3DEPI, an optional preparatory module imparting MT weighting was implemented in the sequence prior to each excitation pulse and used to acquire additional whole brain MT-weighted 3DEPI volumes. MT weighting enhances contrast by saturating the bound pool associated with macromolecular content, which subsequently leads to a reduction in measured signal originating from the free pool due to the process of magnetisation transfer (Wolff and Balaban, 1994). Since a primary source of macromolecular content in the brain is myelin, the greatest signal reduction occurs in white matter (WM), but a negligible reduction in cerebrospinal fluid (CSF), thereby boosting cortical contrast. The MT module consisted of two components: an off-resonance pulse and a spoiler gradient, which spoiled any inadvertent on-resonance excitation.

**Fig. 1:**
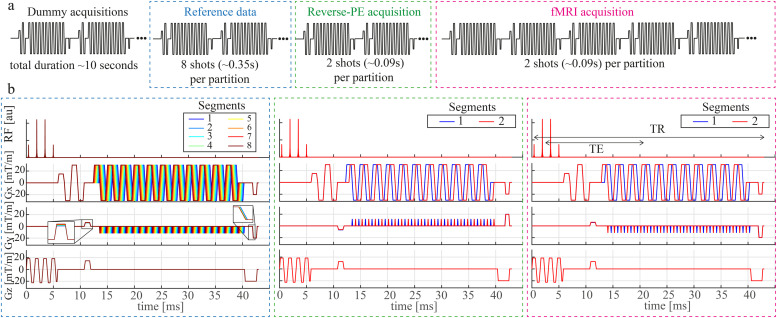
3DEPI sequence diagram with integrated reversed-PE scans. The acquisition is comprised of dummy scans, followed by the acquisition of a fully sampled reference volume (used for estimating coil sensitivities), a single reversed-PE volume, and finally the fMRI acquisition which has the blip gradient polarity restored and matching that of the reference data (a). A single TR from each of these blocks is depicted in (b). These are repeated for each location in the second (slab-selective) phase-encoded direction (denoted “partition” in the Fig.). The fully sampled reference data are acquired with 8 in-plane segments to match the distortions of the fMRI times series, which have 4-fold acceleration and 2-fold segmentation. The colour indicates the temporal order, which is shifted in time so that the k-space centre would always be traversed at the same point in the T2* decay curve. Similar time shifting is applied for the 2-fold segmented under-sampled data. Note that the MT-3DEPI acquisition is achieved by inserting a module consisting of an off-resonance pre-pulse and spoiler gradient to impart MT weighting, using a 1-1 binomial excitation and increasing the coverage in the slab-selective direction (see Table 1 for full details).

The EPI readout was segmented in-plane by a factor of two to reduce susceptibility-induced distortions for both partial coverage fMRI and whole-brain MT-weighted acquisitions. This sequence was also equipped with the capacity to traverse the phase encoding direction of the EPI readout in the opposite direction by reversing the polarity of the phase-encoding “blip” gradient to provide both “blip-up” and “blip-down” volumes for each functional run. The direction of k-space traversal in the readout direction was kept consistent between these volumes. Partial Fourier was enabled in the first PE direction, i.e. in the EPI plane, by reducing the pre-phaser gradient moment and hence skipping the first lines of k-space. The echo time (TE) was identical for “blip-up” and “blip-down” acquisitions, therefore different lines are sampled in the outer part of k-space but for the central portion of k-space, the same lines were acquired regardless of traversal direction. The full sequence diagram for the 3DEPI acquisition is illustrated in Figure 1.

### 2.2 Data acquisition

A 7T MAGNETOM Terra (Siemens Healthcare, Erlangen, Germany) scanner with a head coil equipped with 8 transmit and 32 receiver channels (Nova Medical, Wilmington, USA) was used to collect fMRI data from 20 healthy adult participants (8 males/12 females, age 23 ± 4 years) as part of a task-based study examining how people perceive everyday events. The transmit coil was utilised in a circularly-polarised (CP) mode. All participants gave written informed consent and the study was approved by the University College London Research Ethics Committee. For all participants, two runs of high-resolution BOLD-weighted 3DEPI time-series data were acquired with partial brain coverage, together with an MT-weighted 3DEPI acquisition with whole brain coverage (MT-3DEPI). The imparted MT contrast was found to be sufficient for grey matter (GM) segmentation, while also meeting specific absorption rate (SAR) constraints, when using a Gaussian RF pulse with a flip angle (FA) of 300 degrees, a duration of 4 ms and an off-resonance frequency of 2 kHz. In each of the 3DEPI acquisitions, the first volume was collected with reversed-PE polarity (Posterior-Anterior (PA) direction) relative to the ongoing time series acquisition (Anterior-Posterior (AP) direction). The fMRI protocol used parallel imaging in the slab-selective direction. To ensure that no excited signal from outside of the encoded FOV aliased into the imaging volume, oversampling was employed in this direction by setting the excitation slab thickness to 87% of the encoded field of view. To avoid fat artifact, a water-selective (1-3-3-1 binomial) excitation was used. The sub-pulse interval was 1.5 ms with a time bandwidth product (TBWP) of 24. In the MT-3DEPI protocol, a faster (1-1 binomial) water-selective excitation was used with the same sub-pulse interval and TBWP. The BOLD-weighted 3DEPI and MT-3DEPI protocols had identical echo spacing and readout bandwidth, which were 1.22 ms and 947 Hz/pixel respectively. Owing to in-plane acceleration and readout segmentation, the effective echo spacing in both cases was 152.5 μs, which corresponds to a bandwidth of approximately 27.3 Hz/pixel in the phase encoding direction. Reference data were obtained with a segmented EPI readout (Fig. 1).

B_0_ field-mapping and T_1_-weighted anatomical scans were acquired using dual-echo GE and MP2RAGE (Marques et al., 2010) sequences respectively. The MP2RAGE readout bandwidth was 240 Hz/pixel in the head-foot direction and the phase-encoding was AP. The dual-echo GE excitation was water selective (1-1 binomial sub-pulses with sub-pulse interval corresponding to 1π dephasing between fat and water) and its readout was monopolar with a bandwidth of 500 Hz/pixel. To prepare for B0 map calculation (see Section 2.3.1), the phase difference (Δθ) between the two echoes was calculated by taking the angle of the complex sum over channels of the Hermitian inner product (i.e. $\Delta \theta =∠\sum_{c}S_{2,c}S_{1,c}^{*}$, where S* is the complex conjugate of the measured signal, S, the first subscript denotes echo number, and c indexes the channel number) (Bernstein et al., 1994). The acquisition order was fixed for all sessions (fMRI runs first, followed by MT-3DEPI, B_0_ field mapping, and finally the MP2RAGE). The B_0_ shimming was kept fixed throughout the 3DEPI fMRI runs, whole-brain MT-3DEPI and field map acquisitions. Frequency adjustments were performed prior to each EPI acquisition. Details of all imaging protocols are summarized in Table 1.

**Table 1:** Data acquisition protocols for 3DEPI fMRI, whole brain MT-3DEPI, B_0_ field mapping and structural imaging. (rPE: reversed-PE, seg: segmentation, PF: Partial Fourier).

Scan	Sequence	TR (ms)	TE (ms)	FA	Resolution (mm iso)	PF	Seg. Factor	GRAPPA	Slices	FOV (mm)	Acquisition Time (per vol)	MT	rPE
fMRI runs	3D GE-EPI	44	19.1	15⁰	0.8	6/8	2	4 in PE1 2 in PE2	88	192*192*70	3.872s	No	Yes
Whole-brain MT-3DEPI	3D GE-EPI	100	16.9	8⁰	0.8	6/8	2	4 in PE1 1 in PE2	160	192*192*128	32s	Yes	Yes
B_0_ field map	Dual-echo GE	15	2 / 5.1	7⁰	2	-	1	-	88	220*220*176	2min 27s	-	-
Anatomical Reference	MP2RAGE	5000	2.6	5⁰/3⁰	0.65	6/8	1	3 in PE1 1 in PE2	240	208*208*156	8min 42s	-	-

### 2.3 Data analysis

Data analyses were performed in both “distortion-free” MP2RAGE and distorted EPI spaces. All analysis code is openly available on GitHub (https://github.com/fil-physics/Publication-Code/tree/master/3DEPI-DistortionCorrection).

#### 2.3.1 “Anatomically-faithful” MP2RAGE space

**Fig. 2:**
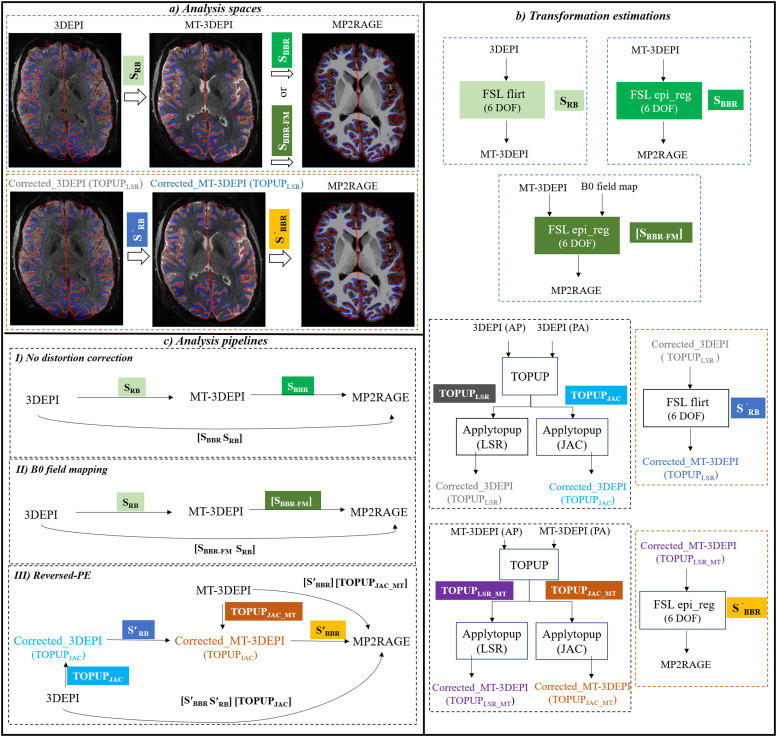
Analysis spaces, transformation estimations and analysis pipelines for no distortion correction, B_0_ field-mapping and reversed-PE distortion correction techniques using 3DEPI and whole-brain MT-3DEPI data. a) Uncorrected and corrected 3DEPI, MT-3DEPI and MP2RAGE spaces and the related transformations used in their processing pipelines. b) Each of the transformation estimations is presented in a separate module indicating input, output data and the related FSL commands. c) Flowcharts of the three pipelines along with their final applied transformations, transforming data from 3DEPI and MT-3DEPI spaces into MP2RAGE space. Note that the analysis code is publicly available on GitHub (https://github.com/fil-physics/Publication-Code/tree/master/3DEPI-DistortionCorrection).

All pre-processing and segmentation steps were performed using FSL (FMRIB, Oxford University) (Jenkinson et al., 2012) and SPM12 (https://www.fil.ion.ucl.ac.uk/spm/) (Acton and Friston, 1998). The processing details for no distortion correction, distortion correction with B_0_ field-mapping data, and distortion correction with reversed-PE data are illustrated in Fig. 2. The pipelines were implemented in MATLAB (version R2020a) using FSL and SPM12 commands. The impact of distortion correction was assessed by comparing the outputs of each of the three pipelines.

In the no distortion correction pipeline (Fig. 2c.I), the first non-reversed-PE 3DEPI volume was registered to the MT-3DEPI volume using rigid-body (RB) registration with the FLIRT algorithm (Jenkinson and Smith, 2001, Jenkinson et al., 2002) employing 6 degrees of freedom (DOF). This produced the transformation matrix S_RB_. The next step was to transform the MT-3DEPI data to the undistorted structural space, defined by the MP2RAGE, using boundary-based registration (BBR) with 6 DOF (Greve and Fischl, 2009) producing the transformation matrix S_BBR_. The resulting transformation matrices, i.e. S_RB_ and S_BBR_, were combined to transform directly from the 3DEPI to the MP2RAGE space with a single spline interpolation.

The B_0_ field-mapping correction pipeline (Fig. 2c.II) augmented the no correction pipeline with the dual-echo GE data. The B_0_ field was estimated from the phase difference between these echoes using the “*fsl_prepare_fieldmap*” command which includes denoising, unwrapping, demeaning, masking, field map extrapolation and unit conversion (to rad/s). Prior to the field estimation step, edge voxels at the periphery of the brain in the phase images were excluded to avoid extrapolating noise into the brain. This was done by creating a mask based on the magnitude image, and eroding this mask using the “*fslmaths -ero*” command. This step is particularly important for the lower resolution field map to exclude all noisy phase voxels around brain edges. The output B_0_ field map was used as an additional input to the “*epi_reg*” command, which provided a mapping from MT-3DEPI to MP2RAGE space (S_BBR-FM_) that incorporated distortion correction. Analogous to the no correction case, S_RB_ and S_BBR-FM_ were combined to transform from the 3DEPI to the MP2RAGE space with a single spline interpolation step.

The reversed-PE correction pipeline (Fig. 2c.III) augmented the no correction pipeline with the reversed phase-encoding volume. Separately for the 3DEPI and MT-3DEPI data, two volumes with opposite phase-encoded directions were used to estimate the B_0_ field using FSL's “*topup*” command. The “*applytopup*” command was used to correct the EPI data twice: either using the least square restoration (LSR) intensity modulation correction method (TOPUP_LSR_) (Morgan et al., 2004) or using the Jacobian (JAC) method (TOPUP_JAC_) (Andersson et al., 2003). LSR offers improved intensity correction and therefore more accurate co-registration. The TOPUP_LSR_ data were therefore used to estimate optimum transformation matrices mapping from 3DEPI to MT-3DEPI distortion-corrected spaces (Sʹ_RB_) and from MT-3DEPI to MP2RAGE distortion-corrected spaces (Sʹ_BBR_). However, LSR requires matched pairs of reversed-PE volumes, which would not be available in typical fMRI time series. Therefore, the JAC intensity modulation correction was used when assessing the performance of the distortion correction methods in a time-series-relevant context. In this case, two spline interpolations were performed, the first via “*applytopup*” and the second following application of the concatenated transformations that map from either the 3DEPI or MT-3DEPI spaces to the MP2RAGE space.

To define GM voxels from the MP2RAGE data (specifically the “UNI” image), the noisy background was removed using the SPM MP2RAGE toolbox (https://github.com/benoitberanger/mp2rage) (O'Brien et al., 2014) and subsequently segmented using unified segmentation (Ashburner, 2009) resulting in a GM probability map. Unified segmentation was also used to segment the MT-3DEPI data in MP2RAGE space. To investigate the impact of distortion correction in different cortical regions, the GM was parcellated using the Harvard-Oxford cortical atlas (Desikan et al., 2006), which was transformed to MP2RAGE space using FSL's nonlinear registration (FNIRT) (Andersson et al., 2007).

#### 2.3.2 Distorted EPI space

To further assess the distortion correction approaches in the distorted EPI space, the MP2RAGE data, along with its extracted GM tissue probability map, were transformed to the distorted MT-3DEPI and 3DEPI spaces using the inverse of the transformations defined in section 2.3.1. The cortical atlas was also transformed into distorted EPI space using the inverse transformations obtained in the no-correction approach (to not bias to one correction scheme over another), and used to parcellate cortical regions in all approaches.

#### 2.3.3 GM tissue boundary mask

For quantitative assessment based on voxel intensities (using the correlation ratio described in section 2.4.2), GM masks focused on boundary regions were defined in both the distorted MT-3DEPI and undistorted MP2RAGE spaces. The GM tissue probability maps, extracted from MT-3DEPI and MP2RAGE data respectively were thresholded (>0.9) and edge detection was applied to these GM masks using the “*fslmaths -edge*” command. The GM edge masks in each space were then dilated using the “*fslmaths -dilF*” command and binarised to create the final, liberal GM boundary masks (see supplementary Fig. S1 for an illustrative example of the GM boundary masks in both spaces).

### 2.4 Evaluation methods and statistics

The Dice coefficient (DC) and correlation ratio (CR) were used to objectively quantify the effectiveness of each distortion correction approach, in both the undistorted MP2RAGE and the distorted EPI spaces.

#### 2.4.1 Dice coefficient

The DC was used to evaluate how well a GM mask defined by the MT-3DEPI ("GM_MT-3DEPI_") data matched the reference GM mask defined by the MP2RAGE data ("GM_MP2RAGE_") as a function of the common probability threshold used to define these masks. The DC varies between zero, meaning no overlap, and one, indicating complete overlap. The DC is calculated by dividing the number of voxels in the intersection of the two binary masks, by the mean number of voxels in both GM_MT-3EPI_ and GM_MP2RAGE_ masks. Our analysis was restricted to GM given the focus on fMRI. A wide range of GM tissue probability thresholds was used to generate the binary masks (0.50 to 0.90 in steps of 0.01). The DC was calculated only for the MT-3DEPI data because the lower GM-WM contrast of the 3DEPI data did not permit robust segmentation.

To calculate the DC in MT-3DEPI space, the MT-3DEPI data were segmented and GM_MP2RAGE_ was transformed into MT-3DEPI space using the inverse transformations (S_BBR_^−1^ for no correction, S_BBR-FM_^−1^ for B_0_ field mapping and Sʹ_BBR_^−1^ for reversed-PE). Note, for the reversed-PE technique, no intensity correction was applied when transforming the GM_MP2RAGE_ probability map to this space. The same range of probability thresholds used for the calculation in MP2RAGE space was again applied when computing the DC in this space.

#### 2.4.2 Correlation ratio

The CR is a quantitative metric that originates from probability theory and has been widely used in the medical image registration literature (Jenkinson and Smith, 2001, Roche et al., 1998) to capture the similarity of two images including that driven by inherent image contrast gradients. The CR is an asymmetric measure that considers nonlinear dependencies between two random variables in comparison to the correlation coefficient, which is a symmetrical linear measure of inter-dependence (Roche et al., 1998). Compared to the DC (which is calculated based on binarized voxel values), the CR metric is sensitive to anatomically-driven contrast variation, e.g. across the boundaries of cortical regions, and therefore to the precision of cortical alignment after distortion correction. The CR is a normalised measure that scales the output between 0 (no correspondence) and 1 (perfect correspondence).

Mutual information (MI) is another well-known measure to assess the performance of two registered images (Maes et al., 2003, Tsai et al., 2004) that has previously been used for quantitative evaluation of distortion correction techniques in 2DEPI (Schallmo et al., 2021). To investigate which measure is more sensitive for our 3DEPI dataset, we used both CR and MI to evaluate two highly-distorted frontal cortical regions, specifically, ventromedial prefrontal cortex (vmPFC) and dorsomedial prefrontal cortex (dmPFC), similar to (Schallmo et al., 2021). This showed the CR to be a more effective measure for quantifying the level of distortion correction (see supplementary Fig. S2 for more detail) and was therefore used in all subsequent analyses.

To calculate the CR in “undistorted” anatomical (i.e. MP2RAGE) space, the GM boundary mask (described in section 2.3.3) was applied to the MP2RAGE data and to the transformed 3DEPI and MT-3DEPI (also now in MP2RAGE space). Cortical parcellation based on the Harvard-Oxford atlas allowed the CR to be computed based on voxel intensities within the GM boundary mask of each ROI separately (see supplementary Fig. S1 top row).

To calculate the CR in distorted EPI space, the MP2RAGE data were first transformed into the MT-3DEPI and 3DEPI spaces using the inverse of the transformations defined as described in Section 2.3.1. Subsequently, the GM boundary mask defined by the MT-3DEPI data was applied to both the MT-3DEPI and transformed MP2RAGE data. Cortical parcellation based on the Harvard-Oxford atlas allowed the CR to be computed based on voxel intensities within the GM boundary mask of each ROI separately, now in MT-3DEPI space (see supplementary Fig. S1 bottom row). Note that for the reversed-PE approach, Jacobian intensity correction was applied when transforming the MP2RAGE data into the MT-3DEPI space.

To calculate the CR in the distorted 3DEPI space, the GM boundary mask of the MT-3DEPI was further transformed into this space using the inverse of the associated transformation (defined as described in Section 2.3.1). Cortical parcellation based on the Harvard-Oxford atlas allowed the CR between the 3DEPI and MP2RAGE data to be computed based on voxel intensities within the GM boundary mask of each ROI.

The relative CR values (CR_rel_) for a given correction scheme in comparison to the no-correction pipeline was given by equation (1). Here k indexes the atlas region, and c the correction method (i.e. reversed-PE or B_0_ field mapping) and was computed based on the mean CR_c_ values in each atlas region. CR_nc_ indicates the mean CR value from the data with no distortion correction applied.

(1) $$CR_{rel}\left(k\right)\;\%=\;\frac{CR_{c}\left(k\right)-CR_{nc}\left(k\right)}{CR_{nc}\left(k\right)}\times 100$$

#### 2.4.3 Statistical analysis

The impact of the distortion correction methods, across all participants, was assessed through a one-way analysis of variance (ANOVA) (Stahle and Wold, 1989) of the CR metric using MATLAB. Between-participant variance was modelled as a random effect, whereas the 3 analysis conditions were modelled as fixed-effects within-participant factors. A p-value <0.05 was deemed significant. The F-statistics quantified the impact the correction schemes had on the CR values. Moreover, to compute pair-wise comparisons among the three approaches, a multiple comparisons test was used, with p-value < 0.05, to detect any significant effect between each pair of pipelines.

## 3 Results

**Fig. 3:**
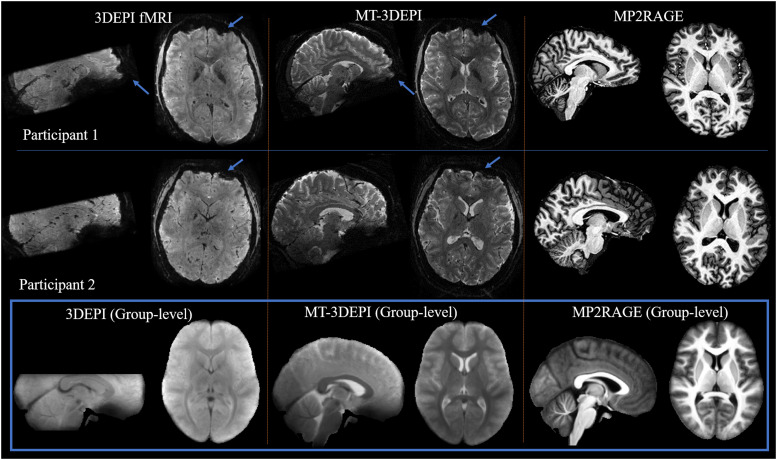
The 3DEPI fMRI, whole brain MT-3DEPI and MP2RAGE data for two representative participants. Similar patterns of distortion can be seen for the 3DEPI fMRI and MT-3DEPI data (e.g. blue arrows) but with the latter having enhanced tissue contrast. The bottom row (blue panel) shows the group level data based on averaging across all 20 participants in MNI_1mm iso space. Note that no distortion correction methods were applied to any of these data.

Exemplar data for two representative participants are shown in Fig. 3. Tissue contrast was substantially improved by adding the MT-weighting module into the 3DEPI sequence. As intended, the distortion patterns were matched between the partial 3DEPI and whole brain MT-3DEPI acquisitions. The last row of this Fig. shows the group mean images in Montreal Neurological Institute (MNI) space (“MNI_1mm iso”) (Horn, 2016).

### 3.1 Effect of MT pre-pulse

**Fig. 4:**
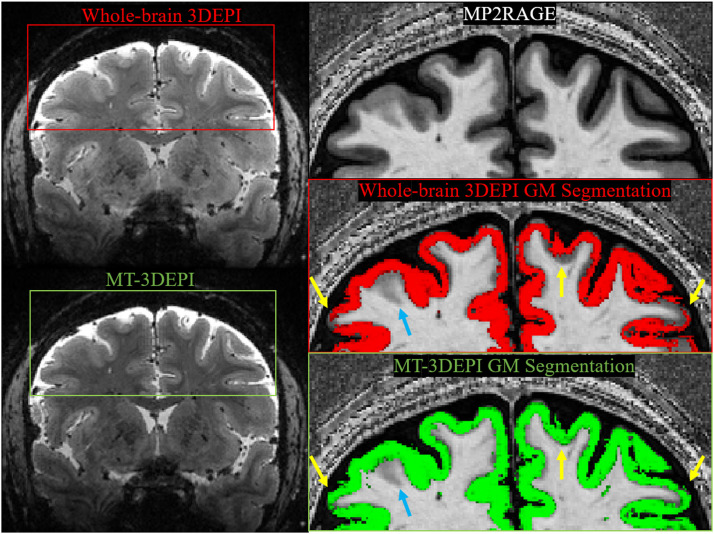
Tissue contrast comparison with and without an MT pre-pulse for the whole brain MT-3DEPI acquisition (all other imaging parameters were identical, but distinct from the 3DEPI fMRI runs – see Table 1). The MT pre-pulse increased contrast (left) and improved the quality of the GM tissue segmentation as evidenced by the greater overlap between the segmentation, with a GM-probability >0.8, and the cortex as defined by the MP2RAGE data (right column). Examples of substantial improvement are indicated by the yellow arrows, while the blue arrows indicate inaccuracies of GM tissue segmentation in both cases.

Two whole-brain EPI images from the same individual with and without the MT pre-pulse module are shown in Fig. 4. Even without the MT module, the contrast was improved relative to the 3DEPI fMRI protocol because of the use of a longer TR and lower flip angle. The contrast, and subsequent cortical segmentations (overlaid on the MP2RAGE image), were further improved by the incorporation of the MT module (yellow arrows) though some errors remained (blue arrows).

### 3.2 Qualitative results of distortion correction

**Fig. 5:**
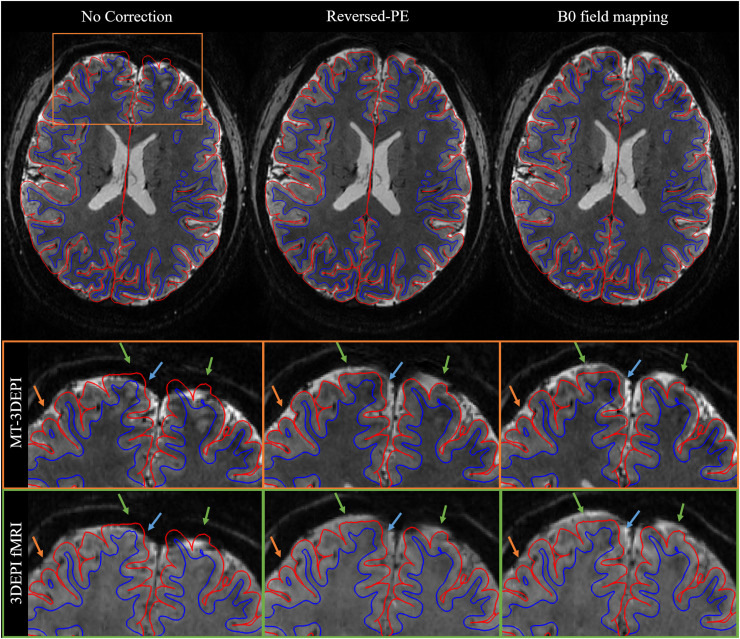
EPI images from a representative participant before and after distortion correction using either the reversed-PE or B_0_ field-mapping approach. The transverse images show the MT-3DEPI data and highlight the region captured by the zoomed view (orange panels). The equivalent anatomical region from the 3DEPI fMRI data is shown in the green panels. Examples of the improvements afforded by both correction techniques (orange arrows), or specifically the reversed-PE method (green arrows), are highlighted. Blue arrows highlight that some degree of misalignment remained after both correction methods. In all cases, the overlaid GM boundaries are derived from the more “anatomically-faithful” MP2RAGE acquisition.

To qualitatively compare the distortion correction methods, we assessed the spatial alignment of the co-registered EPI and MP2RAGE data by visual inspection in MP2RAGE space. Fig. 5 shows the results for one representative participant. For each of the three processing pipelines (Fig. 2), i.e. no distortion correction or correction with either the reversed-PE or B_0_ field mapping approaches, the GM boundaries extracted from the participant's MP2RAGE data were overlaid on the EPI data. Both the B_0_ field-mapping and reversed-PE distortion correction techniques improved cortical alignment of both the whole brain MT-3DEPI and restricted coverage 3DEPI data. The effect of distortion correction is highlighted in the zoomed panels, for both 3DEPI and MT-3DEPI data, in frontal brain regions, where distortions were largest. The improvements were more apparent in the MT-3DEPI images due to their enhanced cortical contrast. Distortions were reduced by both correction methods (orange arrows), but more so by the reversed-PE method (green arrows). Blue arrows indicate where, for both correction methods, some degree of distortion-induced misalignment remained.

### 3.3 Quantitative assessment of distortion correction of whole brain MT-3DEPI data

#### 3.3.1 DC in MP2RAGE space

**Fig. 6:**
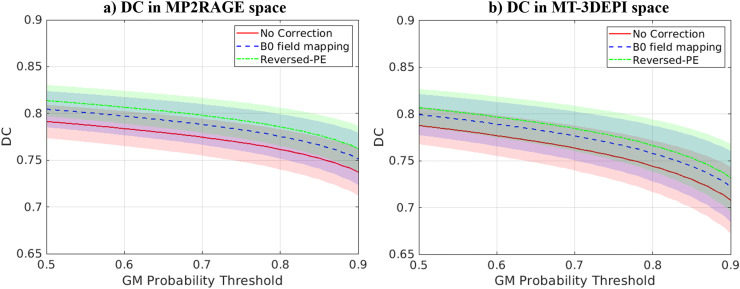
Dice coefficient (DC) between binary GM masks derived from the MP2RAGE and MT-3DEPI data in either MP2RAGE (a) or MT-3DEPI (b) space as a function of GM probability threshold. DC was computed for data without distortion correction (red), and with distortion correction using either the B_0_ field-mapping (blue) or the reversed-PE (green) methods. The bold lines indicate the mean DC across participants, while the shaded areas represent one standard deviation across participants above and below this value.

Fig. 6a shows the DC values, computed in MP2RAGE space, for the uncorrected data, and those corrected with the B_0_ field-mapping and reversed-PE methods as a function of the GM probability threshold used to define the GM mask. The DC increased following distortion correction, indicating more GM overlap between the EPI and MP2RAGE data after correction, and more so for correction by the reversed-PE method. Without correction, the average ± standard deviation DC, across all thresholds and participants, was 0.771±0.021. Following B_0_ field-mapping and reversed-PE based distortion correction, the DC increased to 0.785±0.022 and 0.795±0.019 respectively. After distortion correction, the reversed-PE correction method led to higher DC values than the B_0_ field-mapping approach in 18 out of 20 participants, indicating better overall performance for the reversed-PE method. The DC values were equivalent or higher in only two participants for B_0_ field mapping. The mean DC consistently decreased, for all pipelines, as the GM probability threshold defining the cortex increased, indicating that the more conservative the definition of cortex, the lower the alignment between the MT-3DEPI and MP2RAGE data.

#### 3.3.2 DC in MT-3DEPI space

The DC values computed in the distorted MT-3DEPI space are presented in Fig. 6b. A similar pattern, as a function of probability threshold used to define GM, can be seen relative to the MP2RAGE space analysis. However, the DC values were generally lower in the distorted space, with mean ± standard deviation, across all thresholds and participants, of 0.759±0.025 for no correction, which increased to 0.772±0.027 and 0.780±0.025 for the B_0_ field mapping and reversed-PE correction techniques respectively.

#### 3.3.3 CR in MP2RAGE space

**Fig. 7:**
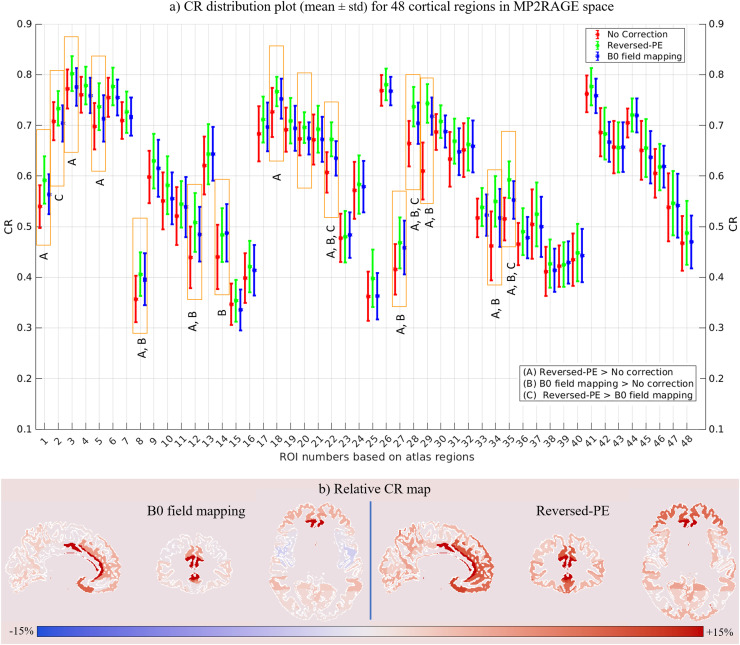
(a) Distribution plots depicting the mean and standard deviation of the CR across participants using the MT-3DEPI data for all cortical ROIs in MP2RAGE space. The results from no-correction, and reversed-PE or B_0_ field-mapping-based distortion correction are illustrated by red, green and blue bars respectively. Those ROIs marked with orange rectangles, selected based on the ANOVA analysis, showed a significant increase in CR following distortion correction. The labels A, B and C indicate significant effects of “reversed-PE > no correction”, “B_0_ field mapping > no correction” and “reversed-PE > B_0_ field mapping” respectively. (b) Relative CR maps, calculated based on equation 1, for either B_0_ field mapping (left) or reversed-PE (right) correction techniques in each of the 48 ROIs defined by the Harvard-Oxford cortical atlas (https://neurovault.org/collections/262/). Red indicates relative CR improvement while blue indicates reduced CR following distortion correction, however the latter effects were never significant.

The CR was computed in the 48 GM regions defined by the Harvard-Oxford cortical atlas. Fig. 7a shows distribution-plots depicting the mean and standard deviation, across participants, of the CR values in each ROI for the data without correction and incorporating correction with either the reversed-PE or B_0_ field-mapping methods.

The one-way ANOVA of these data identified 15 regions (orange rectangles in Fig. 7a) in which distortion correction significantly increased the CR. These regions were predominantly frontal (specifically frontal pole, superior and inferior frontal gyri, subcallosal cortex, cingulate and paracingulate gyri: anterior division), temporal (specifically temporal pole, middle and inferior temporal gyri, para-hippocampal gyrus) and occipital (lateral occipital cortex) cortical regions. The CR values and F-statistics for all identified regions are reported in the “MP2RAGE space” section of Table 2. All but one ROI showed greater improvement with the reversed-PE method, with the largest improvements again observed in frontal and temporal regions. The relative CR maps for the B_0_ field mapping and reversed-PE techniques are presented in Fig. 7b and confirm that the relative CR improvements were mostly in frontal regions, for both correction techniques, but were more widespread and larger for the reversed PE method.

**Table 2:** The mean (µ) and standard deviation (δ), across participants, of the CR values using the MT-EPI data in both MP2RAGE and MT-3DEPI spaces for the Harvard-Oxford cortical ROIs identified by the ANOVA as being significantly increased (p-value < 0.05). F statistics are also presented for these regions (NC: No-correction, rPE: reversed-PE, B_0_: B_0_ field-mapping).

ROI (Name* & Number)		MP2RAGE space				MT-3DEPI space			
		NC (µ ±δ)	rPE (µ ±δ)	B_0_ (µ ±δ)	F-stat	NC (µ ±δ)	rPE (µ ±δ)	B_0_ (µ ±δ)	F-stat
Frontal pole	#1	0.540±0.042	0.592±0.047	0.564±0.040	7.5	-	-	-	-
Insular cortex	#2	0.709±0.038	0.734±0.034	0.704±0.036	4.0	0.673±0.035	0.685±0.034	0.653±0.028	5.0
Superior frontal gyrus	#3	0.772±0.039	0.803±0.035	0.776±0.037	4.1	0.657±0.059	0.702±0.052	0.662±0.051	4.1
Inferior frontal gyrus	#5	0.699±0.046	0.737±0.046	0.714±0.046	3.6	-	-	-	-
Temporal pole	#8	0.358±0.046	0.407±0.043	0.397±0.051	6.0	0.380±0.041	0.431±0.050	0.438±0.050	9.1
Middle temporal gyrus (PD)	#12	0.440±0.061	0.509±0.058	0.485±0.054	7.4	-	-	-	-
Inferior temporal gyrus (AD)	#14	0.441±0.064	0.484±0.053	0.488±0.057	4.1	-	-	-	-
Superior parietal lobule	#18	0.726±0.048	0.767±0.029	0.753±0.040	5.5	0.613±0.054	0.687±0.047	0.647±0.046	11.4
Supramarginal gyrus (PD)	#20	0.674±0.033	0.696±0.030	0.675±0.032	3.2	-	-	-	
Lateral occipital cortex	#22	0.608±0.040	0.673±0.034	0.636±0.034	16.4	0.444±0.044	0.480±0.040	0.465±0.036	3.9
Frontal medial cortex	#25	-	-	-	-	0.527±0.019	0.545±0.028	0.548±0.024	4.3
Juxtapositional lobule cortex	#26	-	-	-	-	0.699±0.042	0.724±0.038	0.693±0.041	3.3
Subcallosal cortex	#27	0.416±0.050	0.469±0.050	0.459±0.053	6.0	0.531±0.017	0.547±0.020	0.545±0.022	3.7
Paracingulate gyrus	#28	0.664±0.045	0.737±0.039	0.705±0.041	15.3	0.579±0.057	0.637±0.049	0.610±0.053	6.0
Cingulate gyrus (AD)	#29	0.610±0.056	0.744±0.038	0.718±0.065	51.0	0.620±0.048	0.673±0.041	0.655±0.038	8.1
Precuneous cortex	#31	-	-	-	-	0.516±0.050	0.558±0.047	0.538±0.041	4.1
Parahippocampal gyrus (AD)	#34	0.462±0.068	0.550±0.050	0.518±0.057	11.4	0.497±0.033	0.524±0.030	0.530±0.030	6.3
Parahippocampal gyrus (PD)	#35	0.515±0.042	0.593±0.036	0.553±0.037	20.1	-	-	-	-
Occipital pole	#48	-	-	-	-	0.398±0.036	0.430±0.030	0.403±0.028	6.2

⁎ Abbreviations: Pars Triangularis (PT), Pars Opercularis (PO), Anterior Division (AD), Posterior Division (PD), Superior Division (SD), Inferior Division (ID), Temporooccipital Part (TP)

Multiple comparison testing identified cortical regions with significant effects of distortion correction. The dominant source of variance was the impact of correction (significantly increased CR in 12 ROIs due to the reversed PE method and in 9 ROIs due to B0 field-mapping). Reversed-PE significantly out-performed B_0_ field mapping correction in 4 ROIs. No ROI had a significant effect of larger CR in the absence of correction. The full results of the ANOVA in MP2RAGE space is provided in supplementary Table S1.

#### 3.3.4 CR in MT-3DEPI space

**Fig. 8:**
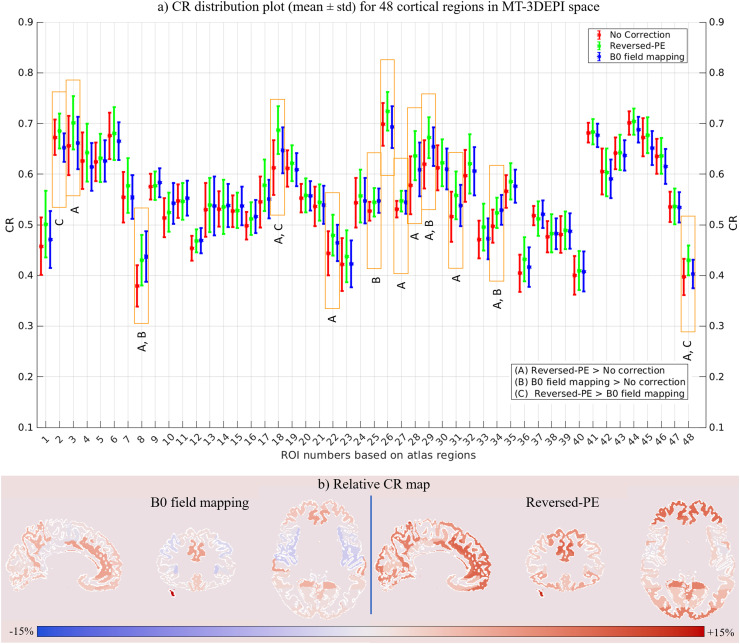
(a) Distribution plots depicting the mean and standard deviation of the CR across participants using the MT-3DEPI data for all cortical ROIs, in the MT-3DEPI space. The results from no-correction, and reversed-PE or B_0_ field-mapping-based distortion correction are shown by red, green and blue bars respectively. Those ROIs marked with orange rectangles, selected based on multiple comparison analysis, showed a significant increase in CR following distortion correction. The A, B and C letters below the orange rectangles indicate “reversed-PE > no correction”, “B_0_ field mapping > no correction” and “reversed-PE > B_0_ field mapping” respectively. (b) Relative CR maps, calculated based on equation 1, for both reversed-PE and B_0_ field mapping correction techniques in 48 ROIs defined by the Harvard-Oxford cortical atlas. Red indicates relative CR improvement while blue indicates reduced CR following distortion correction, however the latter effects were never significant.

The CR was again computed in each of the 48 GM regions of the Harvard-Oxford cortical atlas, but now in the distorted MT-3DEPI space. Fig. 8a shows distribution-plots depicting the mean and standard deviation, across participants, of the CR values in each ROI for the data without correction and incorporating correction with either the reversed-PE or B_0_ field-mapping methods. The CR distributions across the different regions in the MT-3DEPI space followed a similar pattern to the results in the MP2RAGE space, with the reversed-PE distortion correction technique performing best. However, overall, the effect of distortion correction was smaller and was significant in fewer ROIs.

The one-way ANOVA of these data identified 13 regions (orange rectangles in Fig. 8a) in which distortion correction significantly increased the CR. These regions were predominantly frontal (specifically inferior frontal gyrus, frontal medial cortex, subcallosal cortex, cingulate and paracingulate gyri: anterior division), temporal (specifically temporal pole, para-hippocampal gyrus) and occipital (lateral occipital cortex, precuneous cortex, occipital pole) cortical regions. The CR values and F-statistics for all identified regions are reported in the “MT-3DEPI space” section of Table 2.

The relative CR maps for the B_0_ field mapping and reversed-PE techniques are presented in Fig. 8b and confirm that the relative improvements were mostly in frontal regions for both correction techniques, but were more widespread and larger for the reversed-PE method.

Multiple comparison testing identified cortical regions with significant effects of distortion correction. The dominant source of variance was the impact of correction (significantly increased CR in 10 ROIs due to the reversed PE method and in 4 ROIs due to B0 field-mapping). Reversed-PE significantly out-performed B_0_ field mapping correction in 3 ROIs. No ROI had a significant effect of larger CR in the absence of correction. The full results of the ANOVA in MP2RAGE space is provided in supplementary Table S1.

### 3.4 CR of 3DEPI fMRI data in MP2RAGE and 3DEPI spaces

Given the partial brain coverage of the 3DEPI fMRI data, the CR could only be computed in 32 of the 48 regions of the Harvard-Oxford cortical atlas. Supplementary Fig. S3 shows distribution-plots depicting the mean and standard deviation, across participants, of the CR values for each fMRI run following transformation to MP2RAGE space either without correction or incorporating correction with either the reversed-PE or the B_0_ field-mapping method. The results were consistent across runs.

For all pipelines, the CR values were substantially decreased relative to those obtained with the MT-3DEPI data due to the lack of tissue contrast in the 3DEPI data, which makes quantitative comparison much more challenging (also see supplementary Fig. S2).

The one-way ANOVA of these data identified only one region (the posterior division of the middle temporal gyrus) in which distortion correction, specifically with the reversed-PE method, significantly increased the CR. This region was consistently identified in both runs, and had also been identified in the whole brain MT-3DEPI analysis in MP2RAGE space.

The CR values were also calculated in the same 32 ROIs for both fMRI runs in 3DEPI space which is presented in supplementary Fig. S4. Similar to the 3DEPI results in MP2RAGE space, the CR values were reduced substantially in comparison to the MT-3DEPI results. The one-way ANOVA of these data identified two ROIs (temporal pole & frontal orbital cortex) in which there was a significant effect of distortion correction for both correction techniques. The reversed-PE outperformed B_0_ field mapping in the latter ROI. This was consistent across runs.

## 4 Discussion

Here, we compared two approaches to correcting susceptibility-induced image distortions in high resolution gradient echo 3DEPI data to assess their impact in the context of high-resolution fMRI at 7T. The magnetic field inhomogeneity inducing the distortions was estimated either by measurement (B_0_ field-mapping) or modelling (reversed-PE) and subsequently used to undistort the data to improve anatomical fidelity. In a cohort of twenty people, our qualitative and quantitative evaluations revealed that both distortion correction schemes improved correspondence between 3DEPI and MP2RAGE data, in both “distortion-free” MP2RAGE and distorted EPI spaces, albeit to a lesser extent in the latter. The more substantial improvements were consistently obtained using the modelled field, i.e. the reversed-PE approach, as evidenced by higher dice coefficients and correlation ratio improvements. Our regional analyses further demonstrated that the largest benefit of distortion correction, and in particular of the reversed-PE approach, occurred in frontal and temporal brain regions, where susceptibility-induced distortions are known to be greatest.

While distortion correction is a common post-processing step for fMRI data, few studies have directly compared the relative benefit of different correction methods (Holland et al., 2010, Fritz et al., 2014, Schallmo et al., 2021, Hong et al., 2015, Roopchansingh et al., 2020). Those comparisons that have been conducted have demonstrated benefit when using reversed-PE data to perform the distortion correction (Hutton et al., 2002), but have primarily been in the context of DWI (Matakos et al., 2014, Andersson et al., 2003, Morgan et al., 2004) at 3T. Since these DWI studies used a spin-echo readout, which does not suffer from concurrent susceptibility-induced signal dropouts, these findings cannot necessarily be expected to extrapolate to the fMRI context. Signal dropouts can be particularly problematic at 7T due to the shorter T_2_* times and will interact with the EPI readout (e.g. echo spacing, acquisition orientation, resolution) (Volz et al., 2019, Weiskopf et al., 2007) leading to differential manifestation when the phase-encoding gradient polarity is reversed (Mohammadi et al., 2012). Such signal loss could therefore lead to unreliable distortion correction for the reversed-PE approach. Signal dropouts were pronounced in inferior frontal regions, where we observed the greatest differences in the estimated field inhomogeneity, with the model-based approach under-estimating the field inhomogeneity relative to the measured B_0_ field-map (see supplementary Fig. S5). Using a spin-echo acquisition to mitigate dropouts in the context of the current study would prohibit the integrated nature of the blip-reversed volume acquisition, and increase the risk of motion degrading the method's performance (Schallmo et al., 2021). It would also extend the scan time and be more SAR demanding, which would be particularly problematic for the MT-3DEPI approach that is already SAR limited. Only two studies have compared distortion correction techniques at 7T in the context of GE EPI for fMRI (Fritz et al., 2014, Schallmo et al., 2021). They also found that both the B_0_ field-mapping and reversed-PE correction schemes improved anatomical fidelity, and that the benefit was larger with the reversed-PE approach. Although our findings agree with these prior reports, this could not have been assumed a priori, given the very different imaging contexts, most notably the use of slice-selective versus 3D encoding approaches. Furthermore, our exploration of high-resolution fMRI using 3DEPI necessitates a longer EPI readout over which the effect of the distortion-inducing fields accrues.

The benefit of performing functional analyses, including the definition of the cortical surfaces, in the distorted EPI space is to minimise any interpolation and associated smoothing in order to preserve the resolution of the data for subsequent depth-resolved analyses (Polimeni et al., 2018, Chai et al., 2021). In this work, the DC results showed similar trends in both the MP2RAGE and distorted MT-3DEPI spaces, albeit with slightly lower DC values in the latter. The CR results were also comparable across the analysis spaces, but with fewer regions evidencing a significant effect of correction in the distorted EPI spaces. Given that the definition of the cortex is dependent on the data used, as evidenced by the DC results, measures such as mutual information (Schallmo et al., 2021) or CR that are computed within a mask will also be dependent on its definition. This may have contributed to differences in the CR results between the two spaces given that the mask was derived from GM tissue segmentation of either the MT-3DEPI or MP2RAGE data depending on which space the analysis was performed in (see methods), but would not have confounded the comparison across processing pipelines (i.e. with and without distortion correction) where consistent masking was used.

In our study, both distortion correction approaches improved anatomical fidelity, but the reversed-PE approach consistently outperformed B_0_ field mapping in the majority of the cortex (Fig.s 7 & 8) for the two-fold segmented MT-3DEPI data. Further assessments in a phantom and on single-participant data, suggest that this finding would generalise to the more heavily distorted case of non-segmented data (see supplementary Fig. S6, S7 and Table S2). Multiple factors may underlie the differential performance of the B_0_ field mapping and reversed-PE distortion correction approaches. One is that for B_0_ field-mapping the local field inhomogeneity is estimated via phase accrual, which is not a direct measurement of the B_0_ static field. Other tissue-related (e.g. chemical shift, tissue microstructure, flow) and technical (e.g. eddy currents, gradient timing issues) contributions to the phase may not necessarily be identical at each echo time (i.e. may not be removed upon taking the phase difference). Phase maps are also reliant on accurate unwrapping of the phase, which is a notoriously difficult problem that involves a highly nonlinear process, incorporating a binary decision for each voxel regarding the presence or absence of a 2π jump, which is vulnerable to noise in areas where the signal magnitude is low (Andersson et al., 2003). Distortion correction based on B_0_ field-mapping also relies on accurate co-registration, which is hampered by the low contrast of the magnitude images that results from maximising the phase-to-noise ratio via short TEs and imaging at the Ernst angle.

The spatial resolution of the B_0_ field-mapping data was also substantially lower (2 mm versus 0.8 mm voxel length; see Table 1) and may therefore suffer from partial volume effects. However, this resolution was deemed appropriate given the typical smoothness of field inhomogeneity and the need for imaging efficiency and robustness to motion. Increasing the B_0_ field-mapping resolution to the sub-millimetre scale would exacerbate noise and motion sensitivity. By contrast, the reversed-PE technique does not rely on phase-based field estimation and instead estimates the field inhomogeneity that best explains the reversed-PE data given knowledge of how this field should manifest as the phase-encoding direction is reversed (Andersson et al., 2003). Integrating the reversed PE acquisitions into a single run, as we did here, may make the approach more robust to participant motion and co-registration errors. The integrated approach is also very time efficient needing only an additional 3.872 s for the functional runs and 32s for the whole brain acquisitions, whereas the B_0_ field-mapping sequence took 147s. Note that it is possible to use fast imaging methods such as parallel imaging acceleration and/or partial Fourier acquisitions to reduce scan time for B_0_ field mapping, but at the risk of introducing phase errors.

Another potential source of error for B_0_ field mapping is the imprecision of the field estimate at the periphery of the brain due to very rapid phase changes at the brain/scalp/air interfaces. We sought to minimise this issue by eroding the brain mask to exclude affected voxels and then extrapolating the field into these boundary areas. Consistent with this, a phantom-based comparison of reversed-PE based unwarping, without any intensity correction, outperformed B_0_ field mapping around the edges (see supplementary Fig. S8).

Finally, the B_0_ field-mapping approach only corrects for EPI image deformation, but not intensity differences. However, the latter can have a substantial effect, especially in high-resolution imaging (Liu et al., 2021). Intensity correction is not typically performed as part of the B_0_ field mapping distortion correction approach, and was not used in this study, because it is often poorly conditioned. However, a phantom-based evaluation that incorporated intensity correction for both distortion correction methods indicates that combining unwarping with intensity correction leads to greater improvement in the corrected image relative to an undistorted reference (see supplementary Fig. S8) only for the reversed-PE approach, which may partly explain its better performance relative to the B_0_ field mapping approach. In our reversed-PE correction pipeline, both LSR and Jacobian modulations were utilised. The LSR method has previously been shown to compensate for intensity changes owing to stretching/compression more effectively than Jacobian modulation (Andersson et al., 2003) (see also supplementary Fig. S9). However, this modulation technique requires pair-wise scans, not typically available in fMRI, meaning that the Jacobian modulation must be used to correct intensity variations in fMRI time-series. However, to maximise the precision of the overall pipeline in this study, LSR was used on the first two volumes to maximise the accuracy of the co-registration transformation estimation between spaces.

### 4.1 Study limitations

In the context of high-resolution fMRI, 3DEPI offers higher SNR and avoids slice profile effects relative to its 2DEPI counterpart. However, this is at the cost of reduced image contrast, which limits our ability to quantitatively evaluate the impact of distortions and their correction. The lack of contrast prevented reliable GM tissue segmentation of the 3DEPI data meaning that the DC could not be computed for these data, and also lowered the sensitivity of the CR metric, which in essence assesses the degree of overlap of spatial boundaries (see supplementary Fig. S2).

To address the lack of contrast, we implemented an MT-preparation module to acquire whole-brain 3DEPI data with enhanced GM-WM contrast but matched spatial distortions (Chai et al., 2021). As expected (Greve and Fischl, 2009), the co-registration between the partial coverage 3DEPI fMRI and the anatomical MP2RAGE data was improved by using this higher contrast, distortion-matched, whole brain coverage acquisition as an intermediate. The MT-3DEPI acquisition also facilitated more accurate tissue segmentation, and therefore quantitative assessment of the distortion correction performance. However, particularly at 7T, the MT-3DEPI acquisition is SAR limited, necessitating a longer TR and volume acquisition time, and inhomogeneity in the transmit field efficiency results in spatially varying image contrast (supplementary Fig. S10). These factors may affect the GM segmentation of the MT-3DEPI data in cortical regions where the transmit field (B_1_^+^) efficiency, and therefore contrast, is lower but could be addressed with more B_1_^+^-robust solutions that also meet SAR constraints (Chai et al., 2021). Alternative contrast enhancement approaches, such as T_1_-weighted EPI, could be adopted, via an inversion recovery (IR) preparation (van der Zwaag et al., 2018), but are also vulnerable to inhomogeneity in the transmit field efficiency, need a suitable inversion time to be chosen and can suffer from the filtering effect of T_1_ recovery (blurring) over the long EPI blocks.

Both the 3DEPI and MT-3DEPI acquisitions relied upon reference data for unfolding that were obtained with a segmented EPI readout (Fig. 1). In this case, the distortions in the reference data only precisely match the EPI data acquired with the same polarity as the ongoing fMRI data, i.e. there is a mismatch with respect to the blip-reversed volume. As a result, the blip-reversed data had observably lower image quality suggesting that the performance may be further improved with alternative approaches to estimating the sensitivities. For example, FLASH-based reference data would not be biased to one polarity, but would also not be distortion-matched to either polarity. Another point to consider in the 3DEPI data acquisition is the sub-pulse interval within the excitation module. There is a trade-off between the quality of the slab profile and the sub-pulse interval that can be achieved. Given that our protocol used acceleration in the second phase-encoded (slab-selective) direction, it was important that this profile be sufficiently well defined to avoid residual aliasing artefacts. However, the use of an inter-pulse spacing of 1.5ms, which corresponds to 3π dephasing between fat and water, leads to greater signal loss in more off-resonant locations. By contrast, the B0 field mapping used a non-selective excitation allowing greater flexibility on the choice of the inter-pulse interval, which in this case was equivalent to 1π dephasing between fat and water, i.e. approximately 0.5 ms.

This work focused on static geometric distortions in 3DEPI data caused by B_0_ field inhomogeneity. Gradient non-linearity can also lead to spatial localisation errors. In line with recent studies of distortion correction at 7T (Yamamoto et al., 2021), we found that the impact of gradient non-linearity was substantially less than the effect of susceptibility-induced distortions (data not shown) and was therefore not included in the processing pipelines in order to minimise complexity and the need for proprietary information from vendors. However, correcting for gradient non-linearity effects may be of greater benefit in cases where data have been collected from different scanners. For example, in several high-field studies (Schallmo et al., 2021, Yamamoto et al., 2021), structural data were acquired at 3T, but fMRI data at 7T. In addition, participant-induced field changes, e.g. due to movement or breathing, can lead to dynamically varying distortions. Dynamic distortion correction techniques have been developed to address this additional source of error (Hutton et al., 2002, Dymerska et al., 2018, Wallace et al., 2020). However, these methods are not widely utilised in routine fMRI post-processing, e.g. due to the need to access the phase of the functional data or additional navigator-based information and were therefore not included in the present study.

## 5 Conclusions

Both qualitative and quantitative evaluations demonstrated that distortion correction, based on reversed phase-encoded or B_0_ field mapping data, improves the anatomical fidelity of gradient echo 3DEPI data when compared to MP2RAGE data, which are assumed to be “anatomically-faithful”. In the context of high-resolution fMRI at 7T, using reversed-PE based distortion correction has superior performance relative to no correction or the use of B_0_ field mapping across a broad range of cortical regions, particularly in frontal and temporal regions.

## Data and code availability

The data that support the findings of this study are available from e.maguire@ucl.ac.uk upon reasonable request. The analysis code is publicly available in the following repository: (https://github.com/fil-physics/Publication-Code/tree/master/3DEPI-DistortionCorrection).

## CRediT authorship contribution statement

**Vahid Malekian:** Conceptualization, Methodology, Data curation, Software, Formal analysis, Investigation, Visualization, Writing – original draft, Writing – review & editing. **Nadine N Graedel:** Software, Visualization, Writing – review & editing. **Alice Hickling:** Data curation. **Ali Aghaeifar:** Methodology, Software, Writing – review & editing. **Barbara Dymerska:** Methodology, Software, Writing – review & editing. **Nadège Corbin:** Methodology, Software, Writing – review & editing. **Oliver Josephs:** Methodology, Software, Writing – review & editing. **Eleanor A. Maguire:** Funding acquisition, Resources, Project administration, Writing – review & editing. **Martina F. Callaghan:** Conceptualization, Methodology, Investigation, Validation, Supervision, Funding acquisition, Resources, Project administration, Writing – original draft, Writing – review & editing.

## Declaration of Competing Interest

The authors declare that they have no conflict of interest.

## Appendix Supplementary materials

Image, application 1
