# Supplementary material for: Mitigating susceptibility-induced distortions in high-resolution 3DEPI fMRI at 7T
Source: Neuroimage. 2023 Oct 1;279:120294. doi: 10.1016/j.neuroimage.2023.120294 (PMC10951962; doi:10.1016/j.neuroimage.2023.120294)
Supplement: Supplementary file 1 [file mmc1.docx]

**Supplementary Materials**


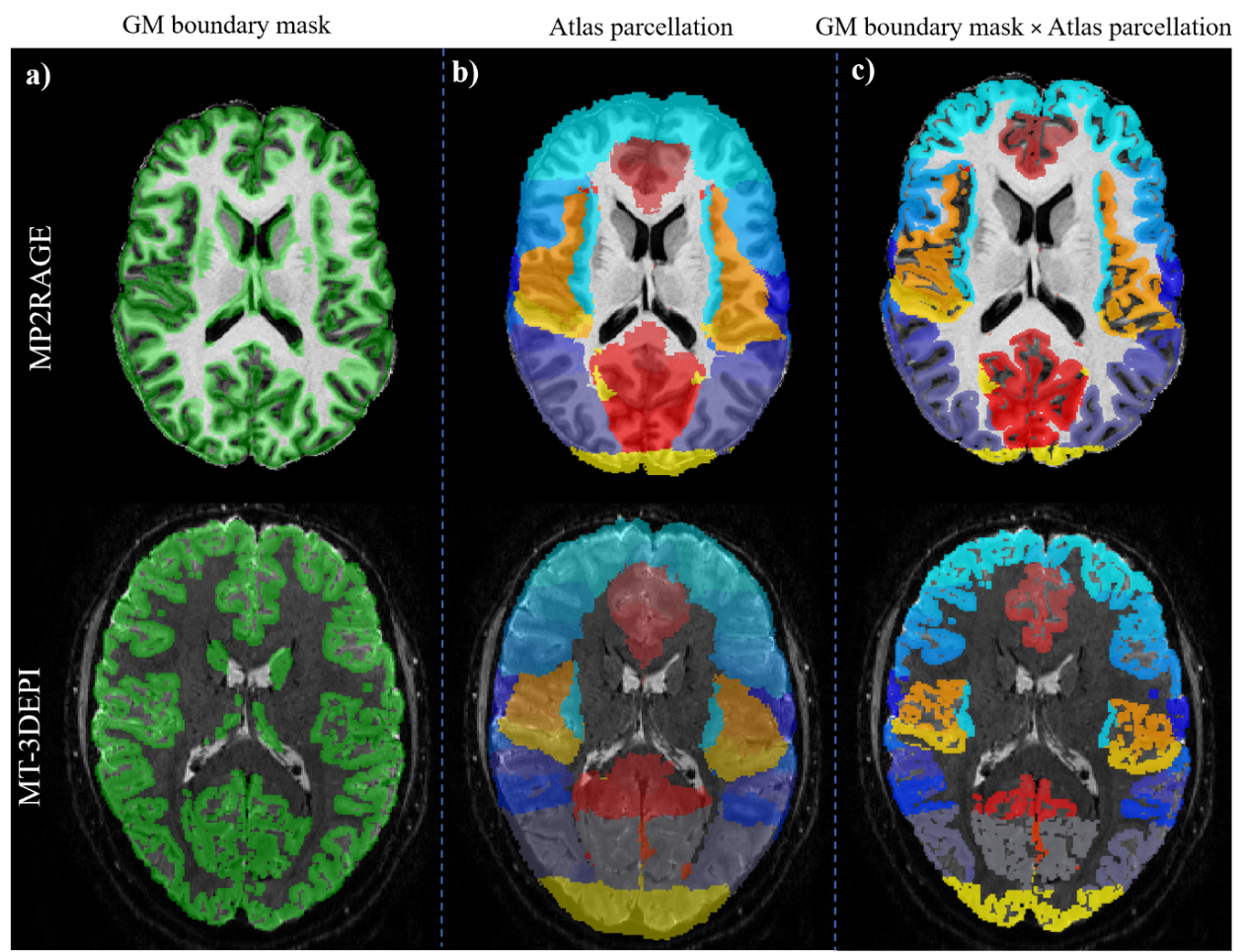


Figure S1. Illustrative example of the GM boundary masks (a), atlas parcellation (b) and final parcellated GM boundary masks (c) in both MT-3DEPI (top raw) and MP2RAGE spaces (bottom raw). Note that Harvard-Oxford atlas was used with 48 cortical regions.


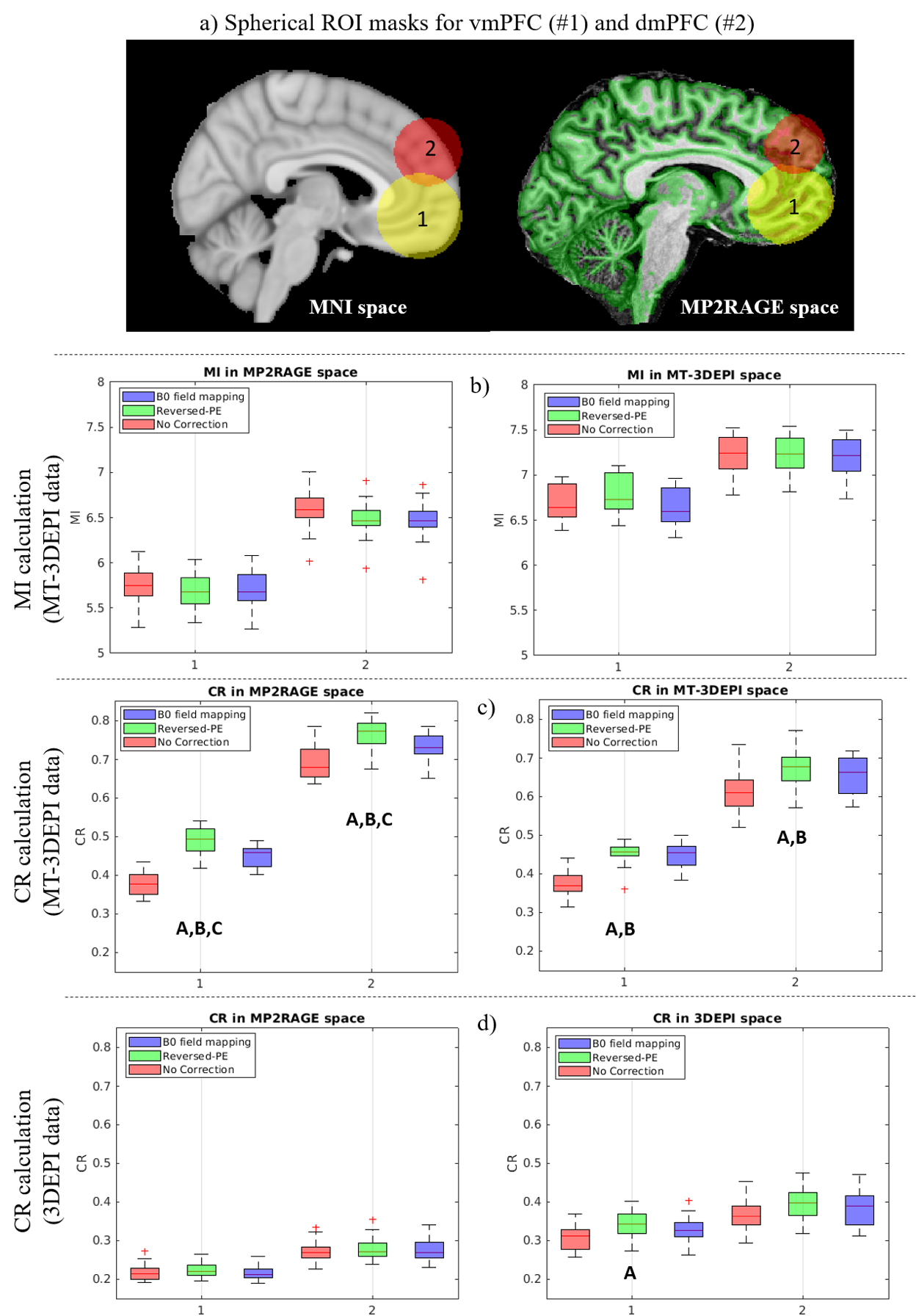


Figure S2. Quantitative assessments of distortion correction methods in two frontal brain regions using mutual information (MI) and correlation ratio (CR) for MT-3DEPI and 3DEPI fMRI data. a) The vmPFC (radius = 26mm and MNI coordinate = [-1.26,48.08, -8.07]) and dmPFC (radius = 21mm and MNI coordinate = [1.26,53.14, 29.88]) ROIs were defined similar to [1], in MNI space and transformed to the MP2RAGE space. (GM boundary mask is shown in green is overlaid on the MP2RAGE data with which it was defined). b) The MI boxplots in vmPFC (ROI #1) and dmPFC (ROI #2) for the MT-3DEPI data in MP2RAGE (left) and MT-3DEPI (right) spaces. c) The CR boxplots in vmPFC and dmPFC for the MT-3DEPI data in MP2RAGE and MT-3DEPI spaces. d) The CR boxplots in vmPFC and dmPFC for the 3DEPI data in MP2RAGE and 3DEPI spaces. Note that A, B and C letters in the plots indicate the following significant differences “reversed-PE > no correction”, “B0 field mapping > no correction” and “reversed-PE > B0 field mapping” respectively.


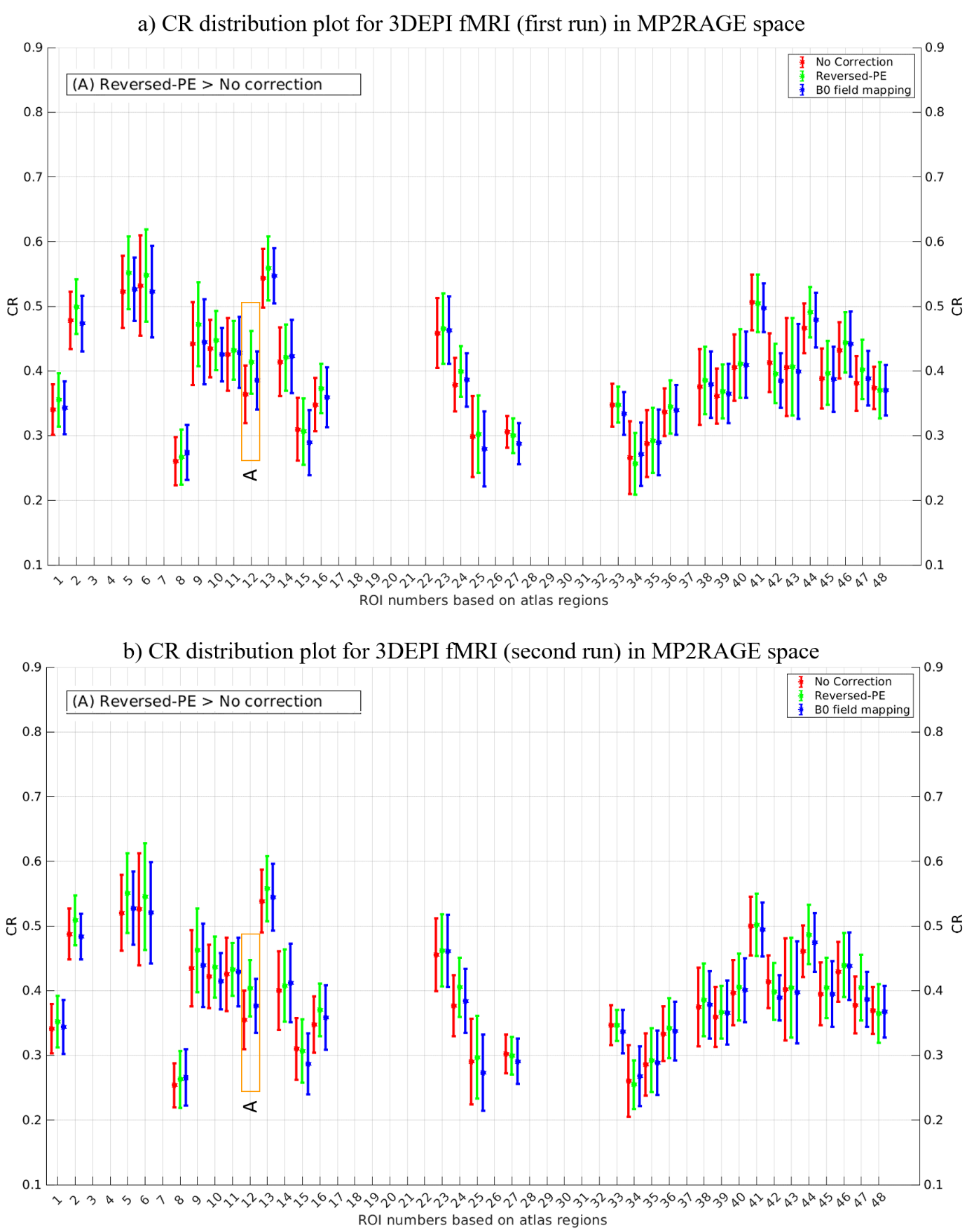


Figure S3. The CR distribution plots (mean ± std) using the 3DEPI fMRI data for 32 cortical ROIs of the first (a) and second (b) fMRI runs in MP2RAGE space. The no-correction, reversed-PE and B0 field mapping techniques are shown in red, green and blue respectively. The orange boxes highlight the one cortical ROI with significantly increased CR following distortion correction, which occurred for the case of “reversed-PE > no correction”. The CR distributions were consistent across runs.


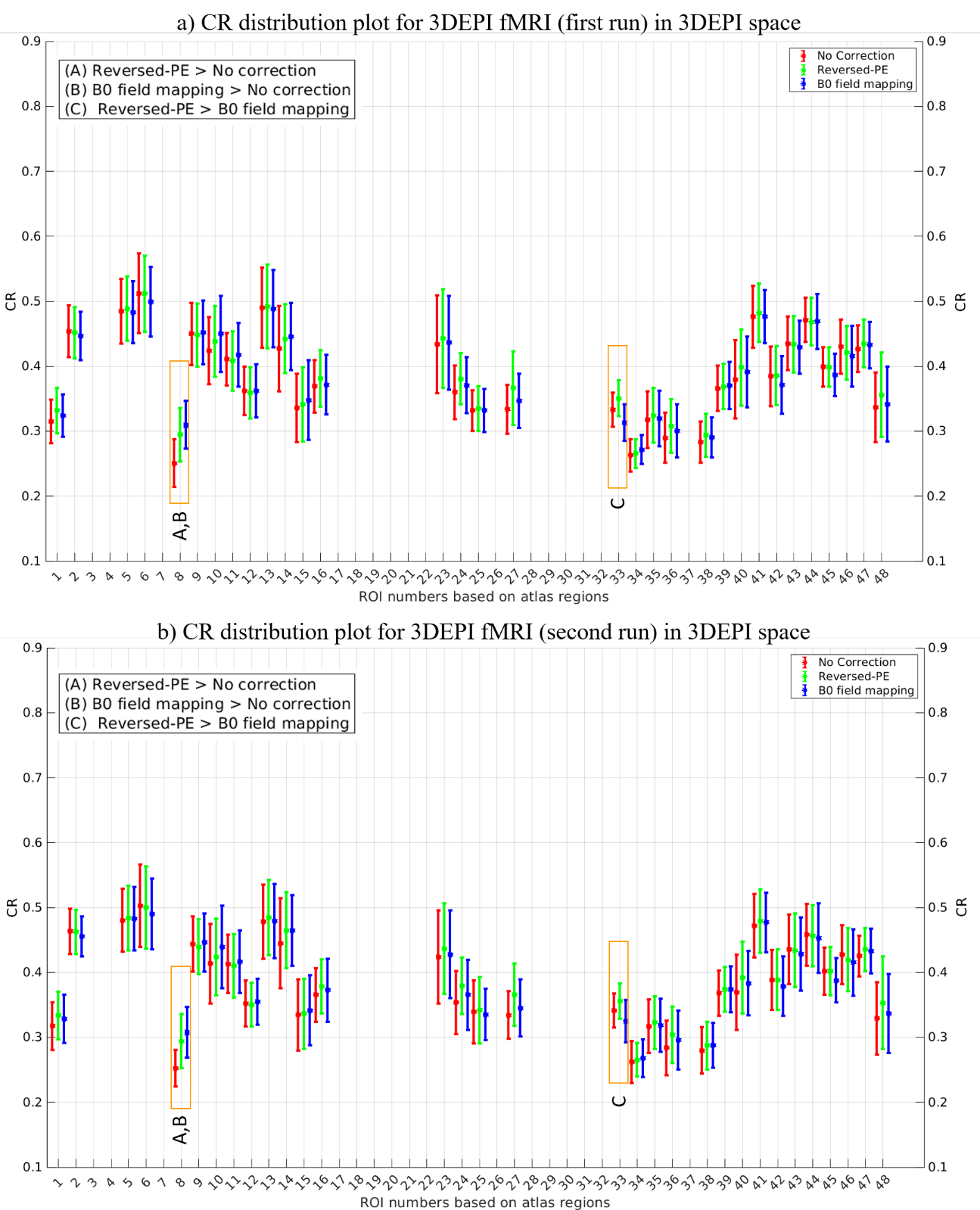


Figure S4. The CR distribution plots (mean ± std) using the 3DEPI fMRI data for 32 cortical ROIs of the first (a) and second (b) fMRI runs in 3DEPI space. The no-correction, reversed-PE and B0 field mapping techniques are shown in red, green and blue respectively. The orange boxes highlight the two cortical ROIs with significantly increased CR following distortion correction. The A, B and C letters below the orange rectangles indicate “reversed-PE > no correction”, “B0 field mapping > no correction” or “reversed-PE > B0 field mapping” respectively. The CR distributions were consistent across runs.


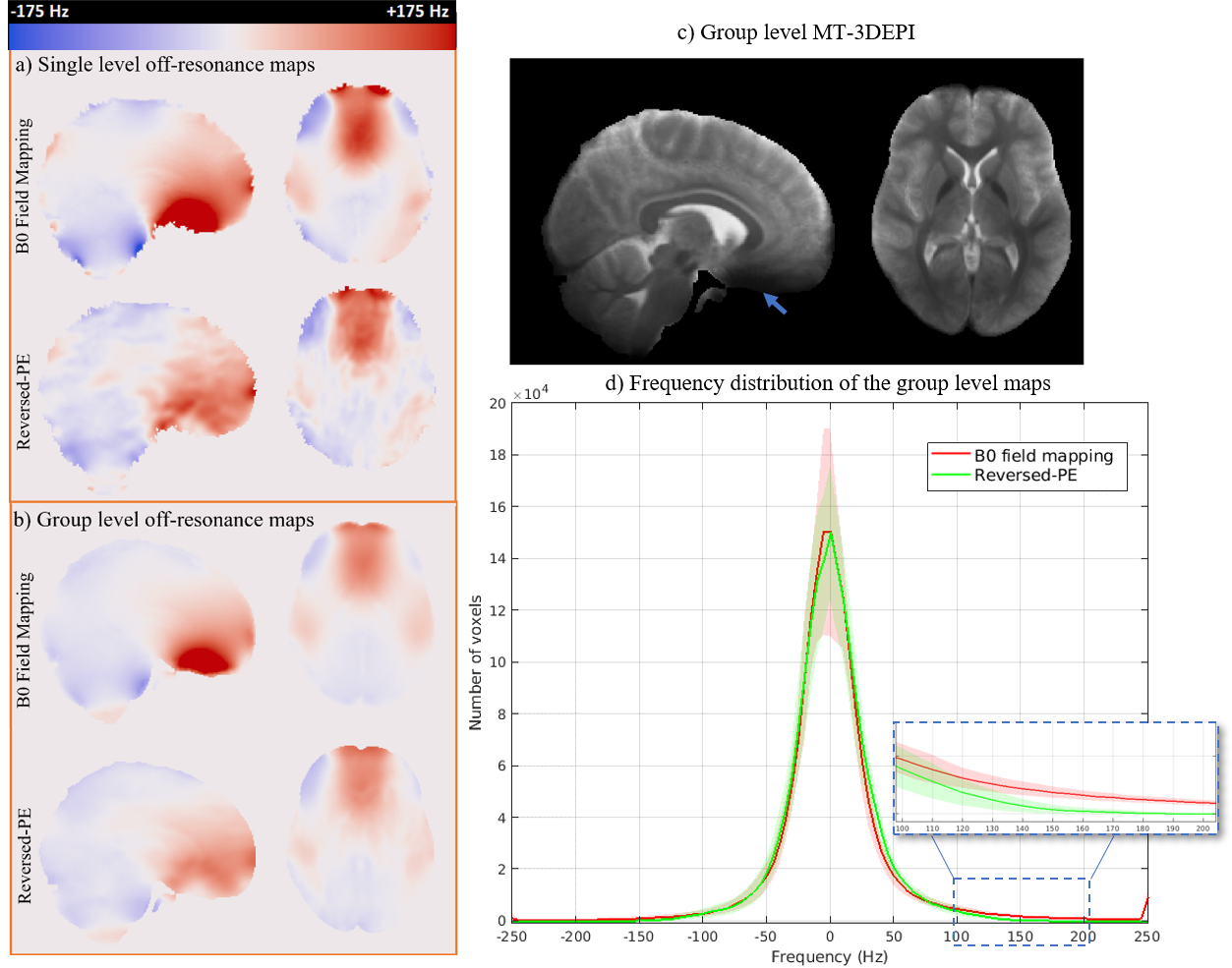


Figure S5. The single-level (a) and group-level (b) off-resonance maps estimated by both the B0 field-mapping and reversed-PE methods. The uncorrected whole brain MT-3DEPI data averaged across the cohort (c). The blue arrow indicates susceptibility-induced signal loss in the inferior frontal cortex of the averaged MT-3DEPI data. (d) Frequency distribution of the fields estimated with the B0 field-mapping (red) and reversed-PE (green) methods. The mean and standard deviation across participants are depicted by solid coloured lines and shaded areas respectively. The zoomed panel in (d) shows the higher off-resonance frequencies estimated by B0 field mapping. This correspond spatially to inferior frontal cortex. The average root mean square values are 34.4 Hz and 46.9 Hz for reversed-PE and B0 field-mapping methods respectively.


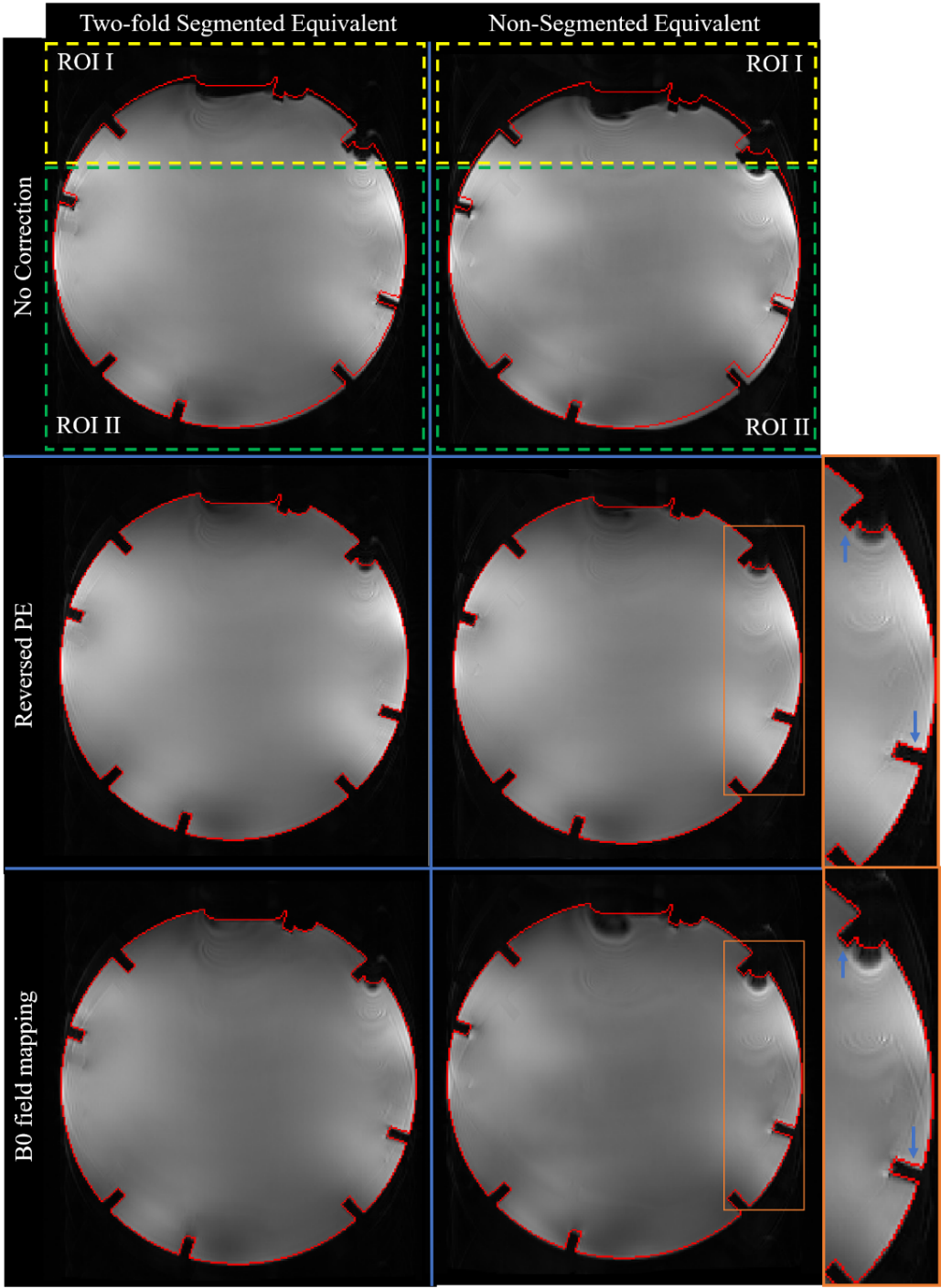


Figure S6. Differentially segmented 3DEPI images from a phantom before (top row) and after distortion correction using either a reversed-PE (middle row) or B0 field-mapping (bottom row) approach. The red boundary was defined by a “distortion-free” reference gradient echo acquisition and overlaid on the 3DEPI data to ease visual inspection. More heavily segmented 3DEPI data had less distortion due to the shorter effective echo-spacing (left column). In line with our *in vivo* observations, both correction techniques reduced the effects of susceptibility-induced distortions, with the reversed-PE correction method outperforming B0 field mapping at the phantom edges, particularly for the less segmented data (see blue arrows in the enlarged orange panels). The quantitative measure (DC) is reported in Table S2 for two ROIs (demarked by dashed yellow and green rectangles) in both 3DEPI datasets using the three approaches. Note that while the protocol used to acquire this phantom data largely matched that of the MT-3DEPI protocol used in the main manuscript, the use of parallel imaging was avoided to maximise image quality and SNR. Instead, segmentation factors of either 4 or 8 were used to achieve the identical echo spacing of an acceleration factor of 4 combined with either non-segmented or two-fold segmentation respectively. The lesser segmentation required a longer TE of 25 ms instead of 16.9 ms. The spatial resolution of the B0 field mapping phantom data matched that of the 3DEPI acquisitions (i.e. was 0.8 mm isotropic resolution). For the phantom analysis, ‘epi_reg’ was not used because there is no GM-WM contrast in the phantom images. Instead, ‘fugue’ was used to unwarp the EPI data using a field map, prepared by the ‘fsl_prepare_fieldmap’ command.


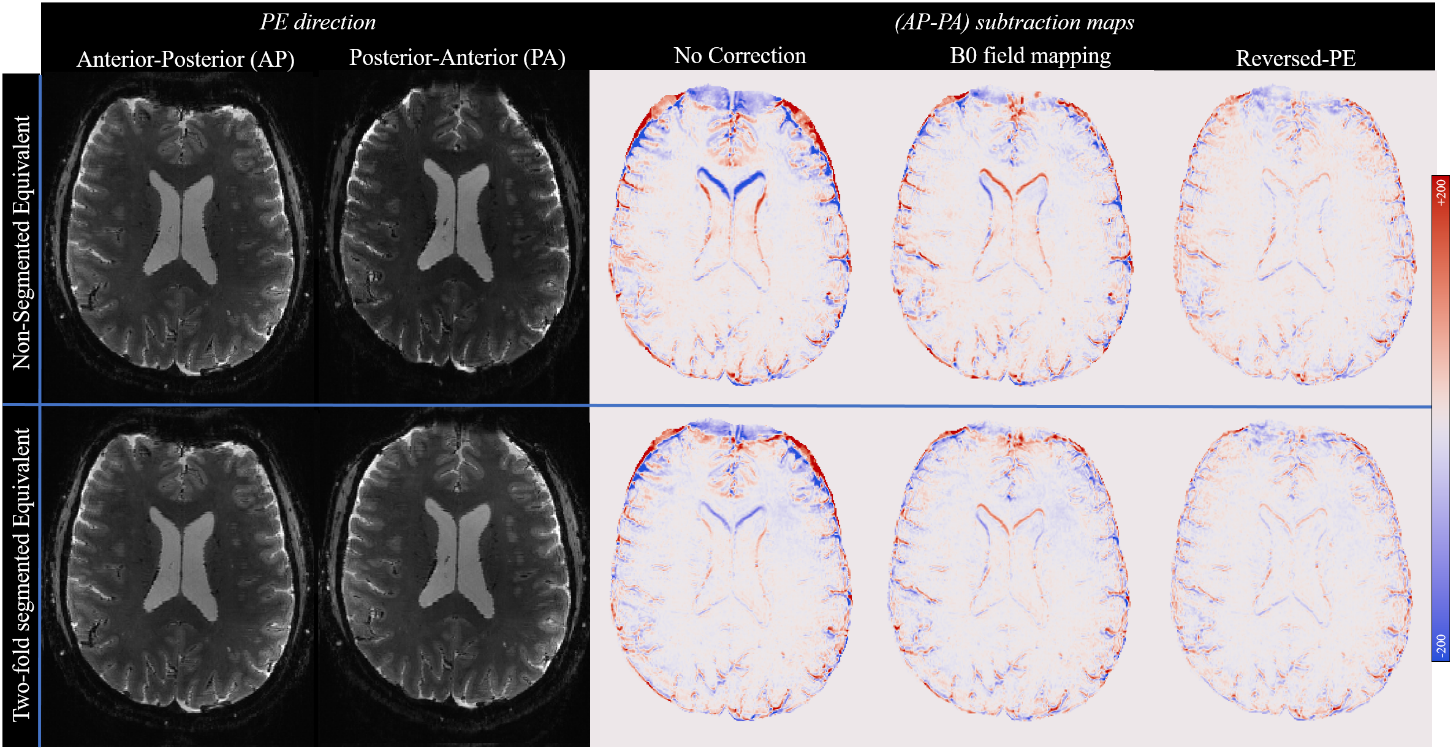


Figure S7. Exemplar *in vivo* differentially segmented MT-3DEPI images acquired with both anterior-posterior (AP) and posterior-anterior (PA) phase encoding direction along with the AP-PA subtraction maps for the cases of no correction, or distortion correction with either the B0 field mapping or reversed-PE correction method. It is apparent that higher segmentation and distortion correction both help in reducing differences between AP and PA images. In addition, the reversed-PE method outperformed the B0 field mapping in decreasing the residuals in the subtraction maps regardless of segmentation factor. This provides further support for the conclusion that our findings will generalise to the more common case of non-segmented, but accelerated, data. Note that the same protocols were used to acquire these data as in the phantom data of Figure S6.


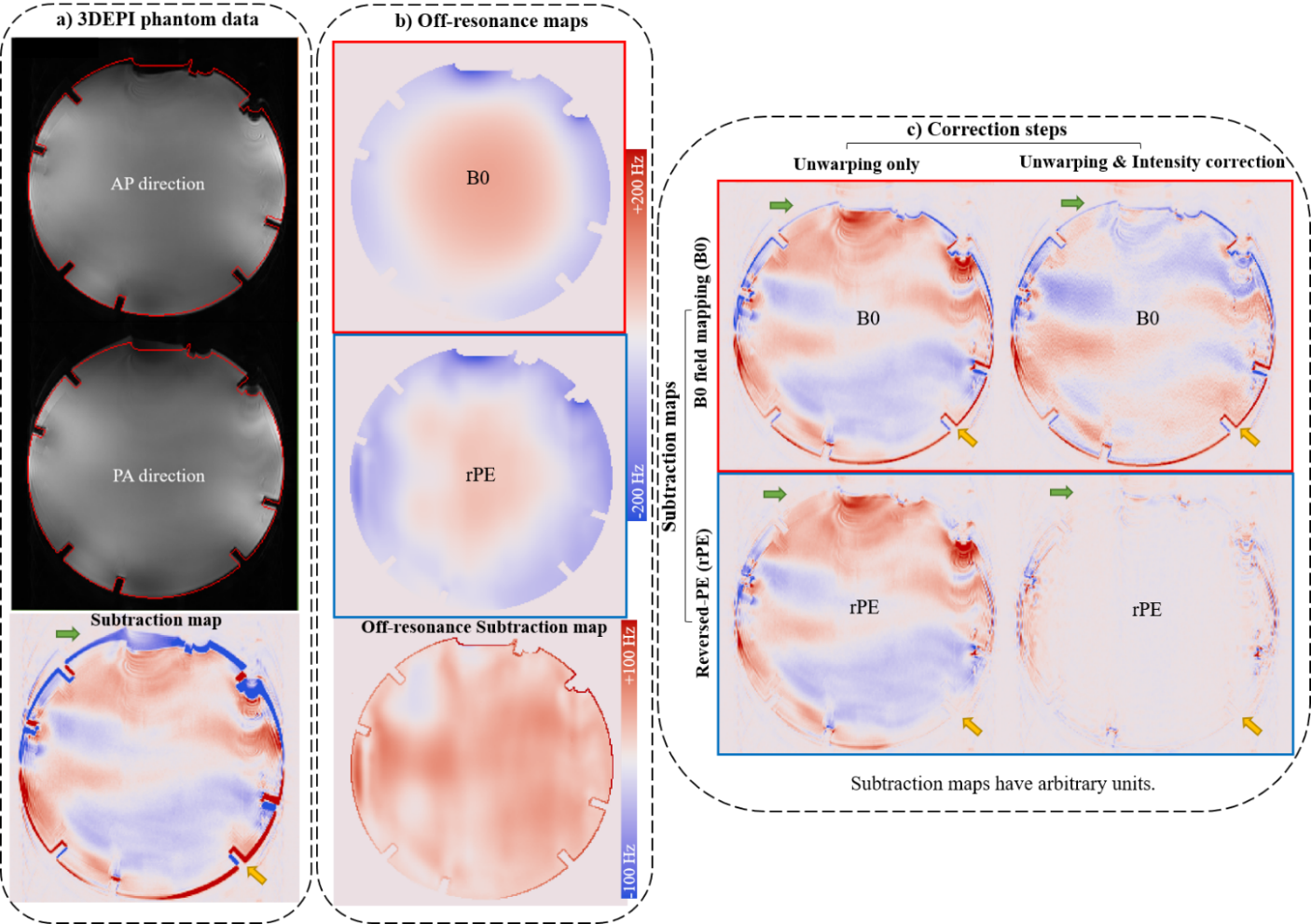


Figure S8. Two-fold segmented equivalent 3DEPI phantom images with anterior-posterior (AP) and posterior-anterior (PA) PE directions along with the difference between the AP and PA images prior to correction (a). The phantom off-resonance maps estimated by the B0 field mapping with matched resolution (i.e. 0.8 mm isotropic) and reversed-PE correction methods. Qualitative inspection indicates global correspondence between the B0 field mapping-based estimate and the field map obtained from reversed-PE data, but with local differences visible, particularly to the left of centre. The B0 and reversed-PE off-resonance maps were subtracted to better visualise these differences. To disentangle the impact of the sub-steps of the distortion correction, the differences between volumes acquired with AP and PA phase encoding were compared (across methods) after unwarping only (c, first column) and unwarping with intensity correction (c, second column). Without correction, the difference between the AP and PA volumes is pronounced, particularly around the periphery of the phantom (indicated by arrows) where there is notable expansion/contraction depending on the phase-encoding direction. This effect is reduced by distortion correction, particularly for the reversed-PE approach. For the B0 field mapping approach, intensity correction was performed using the fugue command with the “--icorr” flag, while the reversed-PE method used Jacobian modulation. The correspondence between the AP and PA data is much greater following intensity correction using the reversed-PE method (c, second column), but remains on a par with unwarping only for the B0 field mapping approach. It is worth mentioning that integrating intensity correction into the pipeline for B0 field-mapping-based correction was only performed for completeness. It was not used in the main pipeline because the step is often poorly conditioned for the B0 field mapping approach leading to inaccuracies in regions of susceptibility-induced magnetic field inhomogeneity. Note that this is the same data as used in Figure S6.


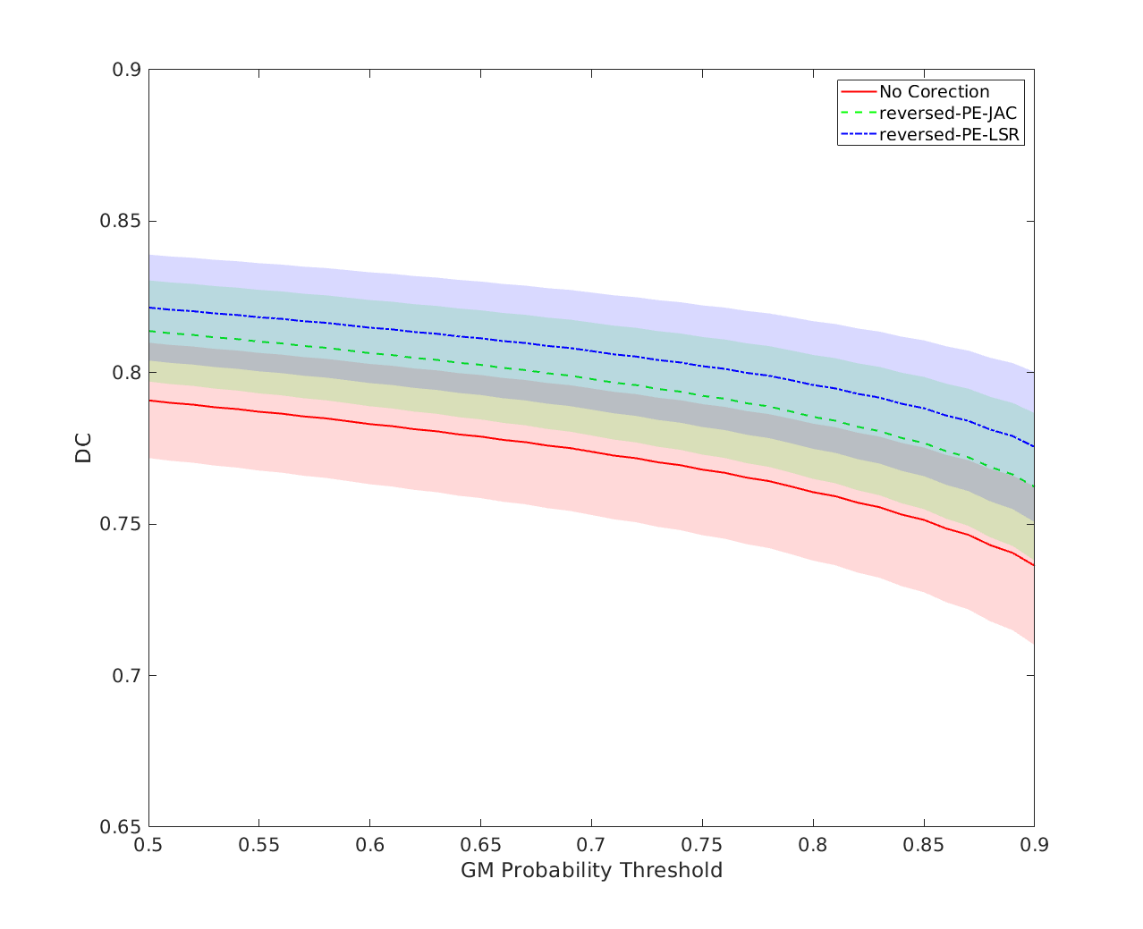


Figure S9. Dice coefficient between binary GM masks derived from the MP2RAGE and MT-3DEPI data in MP2RAGE space using a wide range of GM probability thresholds without distortion correction (red), and with reversed-PE based distortion correction using either LSR (blue) or Jacobian (green) intensity correction.


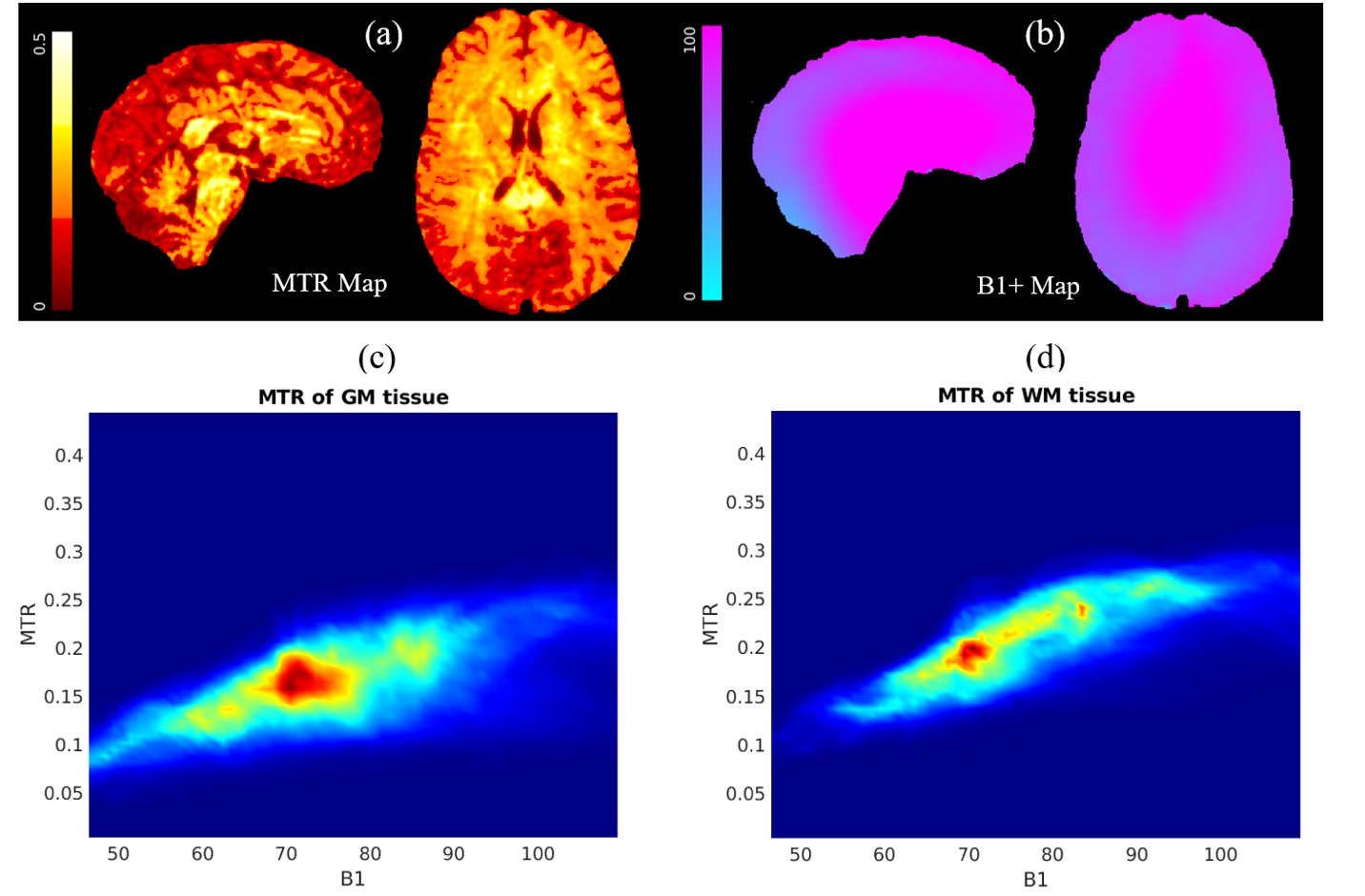


Figure S10. Magnetisation Transfer Ratio (MTR) map (a) calculated as the difference between 3DEPI data obtained with and without an MT pre-pulse relative to the 3DEPI data without an MT pre-pulse, and the corresponding B1+ map (b). The pattern of MTR inhomogeneity across the brain matches that of the B1+ efficiency. Density plots of MTR versus B_1_+ in GM (c) and WM (d) voxels show that these measures are highly correlated with a larger MTR coinciding with higher B_1_^+^ efficiency. Generally higher MTR values are seen in WM, for equivalent power, due to the higher macromolecular content.

Table S1. Multiple comparison analysis between correction techniques using MT-3DEPI data in both MP2RAGE and MT-3DEPI spaces. Overlapped ROIs with p-values < 0.05 in both spaces are shown in yellow. ROIs with p-values < 0.05 in only one space are highlighted in blue. The p-values of all cortical regions are reported for each comparison test. NC, rPE and B0 stand for no correction, reversed-PE and B0 field-mapping methods respectively.

| **#** | **ROI Name*** | **MT-3DEPI**  **(MP2RAGE space)** | | | **MT-3DEPI**  **(MT-3DEPI space)** | | |
| --- | --- | --- | --- | --- | --- | --- | --- |
|  |  | **NC vs rPE**  **(A)** | **NC vs B0**  **(B)** | **rPE vs B0**  **(C)** | **NC vs rPE**  **(A)** | **NC vs B0**  **(B)** | **rPE vs B0**  **(C)** |
| 01 | frontal pole | 0.0008 | 0.1833 | 0.1027 | 0.0625 | 0.7663 | 0.2485 |
| 02 | insular cortex | 0.0717 | 0.9224 | 0.0290 | 0.4775 | 0.1337 | 0.0079 |
| 03 | superior frontal gyrus | 0.0307 | 0.9467 | 0.0649 | 0.0289 | 0.9496 | 0.0603 |
| 04 | middle frontal gyrus | 0.2532 | 0.9898 | 0.1999 | 0.6089 | 0.7624 | 0.2306 |
| 05 | inferior frontal gyrus, PT | 0.0256 | 0.5386 | 0.2483 | 0.8346 | 0.9926 | 0.8913 |
| 06 | inferior frontal gyrus, PO | 0.1727 | 0.9993 | 0.1613 | 0.9546 | 0.7371 | 0.5569 |
| 07 | precentral gyrus | 0.3604 | 0.8091 | 0.7323 | 0.3136 | 0.9999 | 0.3212 |
| 08 | temporal pole | 0.0048 | 0.0299 | 0.7818 | 0.0030 | 0.0007 | 0.8837 |
| 09 | superior temporal gyrus, AD | 0.1655 | 0.5726 | 0.6863 | 0.9682 | 0.6070 | 0.7561 |
| 10 | superior temporal gyrus, PD | 0.1932 | 0.9597 | 0.3060 | 0.6611 | 0.0662 | 0.3405 |
| 11 | middle temporal gyrus, AD | 0.3752 | 0.5541 | 0.9497 | 0.9967 | 0.8718 | 0.8330 |
| 12 | middle temporal gyrus, PD | 0.0011 | 0.0414 | 0.4010 | 0.1405 | 0.1273 | 0.9986 |
| 13 | middle temporal gyrus, TP | 0.4208 | 0.4050 | 0.9996 | 0.8506 | 0.8974 | 0.9946 |
| 14 | inferior temporal gyrus, AD | 0.0581 | 0.0326 | 0.9681 | 0.9512 | 0.8753 | 0.9801 |
| 15 | inferior temporal gyrus, PD | 0.8619 | 0.6612 | 0.3527 | 0.9901 | 0.6377 | 0.7206 |
| 16 | inferior temporal gyrus, TP | 0.3317 | 0.5854 | 0.8988 | 0.3266 | 0.1599 | 0.9085 |
| 17 | postcentral gyrus | 0.1768 | 0.6388 | 0.6425 | 0.0706 | 0.9129 | 0.1633 |
| 18 | superior parietal lobule | 0.0052 | 0.09669 | 0.4846 | 0.0000 | 0.0793 | 0.0341 |
| 19 | supramarginal gyrus, AD | 0.4203 | 0.9731 | 0.5531 | 0.6292 | 0.9713 | 0.4871 |
| 20 | supramarginal gyrus, PD | 0.0716 | 0.9926 | 0.0922 | 0.8564 | 0.8784 | 0.9989 |
| 21 | angular gyrus | 0.3657 | 0.9985 | 0.3935 | 0.7975 | 0.9666 | 0.9181 |
| 22 | lateral occipital cortex, SD | 0.0000 | 0.0455 | 0.0053 | 0.0193 | 0.2508 | 0.4679 |
| 23 | lateral occipital cortex, ID | 0.9837 | 0.9239 | 0.9768 | 0.5781 | 0.9964 | 0.6286 |
| 24 | intracalcarine cortex | 0.7889 | 0.9040 | 0.9714 | 0.6545 | 0.9623 | 0.8117 |
| 25 | frontal medial cortex | 0.0759 | 0.9998 | 0.0789 | 0.0620 | 0.0231 | 0.9123 |
| 26 | juxtapositional lobule cortex | 0.4280 | 0.9920 | 0.3618 | 0.1231 | 0.9106 | 0.0501 |
| 27 | subcallosal cortex | 0.0054 | 0.0269 | 0.8249 | 0.0391 | 0.0839 | 0.9409 |
| 28 | paracingulate gyrus | 0.0000 | 0.0096 | 0.0421 | 0.0028 | 0.1638 | 0.2449 |
| 29 | cingulate gyrus, AD | 0.0000 | 0.0000 | 0.1774 | 0.0000 | 0.0305 | 0.3781 |
| 30 | cingulate gyrus, PD | 0.1308 | 0.9945 | 0.1587 | 0.7406 | 0.9879 | 0.6495 |
| 31 | precuneous cortex | 0.0619 | 0.6140 | 0.3641 | 0.0154 | 0.2937 | 0.3659 |
| 32 | cuneal cortex | 0.7803 | 0.8992 | 0.9705 | 0.3477 | 0.8538 | 0.6652 |
| 33 | frontal orbital cortex | 0.1984 | 0.8791 | 0.4243 | 0.1512 | 0.9908 | 0.1925 |
| 34 | parahippocampal gyrus, AD | 0.0000 | 0.0110 | 0.2058 | 0.0224 | 0.0045 | 0.8312 |
| 35 | parahippocampal gyrus, PD | 0.0000 | 0.0082 | 0.0057 | 0.1637 | 0.6058 | 0.6496 |
| 36 | lingual gyrus | 0.1770 | 0.6266 | 0.6551 | 0.0784 | 0.5948 | 0.4396 |
| 37 | temporal fusiform cortex,AD | 0.6017 | 0.9663 | 0.4496 | 0.7859 | 0.9337 | 0.5699 |
| 38 | temporal fusiform cortex, PD | 0.5478 | 0.9816 | 0.6626 | 0.8013 | 0.8455 | 0.9962 |
| 39 | temporal occipital fusiform cortex | 0.9710 | 0.8451 | 0.9444 | 0.7475 | 0.8233 | 0.9902 |
| 40 | occipital fusiform gyrus | 0.6944 | 0.8841 | 0.9373 | 0.7399 | 0.8348 | 0.9845 |
| 41 | frontal operculum cortex | 0.4170 | 0.9345 | 0.2446 | 0.9695 | 0.7370 | 0.5901 |
| 42 | central opercular cortex | 0.9849 | 0.4094 | 0.5066 | 0.9907 | 0.5496 | 0.6311 |
| 43 | parietal operculum cortex | 0.9994 | 0.9998 | 0.9999 | 0.9875 | 0.9110 | 0.8404 |
| 44 | planum polare | 0.2611 | 0.2960 | 0.9967 | 0.9340 | 0.2387 | 0.1242 |
| 45 | heschls gyrus | 0.9586 | 0.7265 | 0.5543 | 0.9306 | 0.1476 | 0.0695 |
| 46 | planum temporale | 0.6501 | 0.6237 | 0.9990 | 0.9963 | 0.1854 | 0.1591 |
| 47 | supracalcarine cortex | 0.9132 | 0.9869 | 0.9657 | 0.9957 | 0.9937 | 0.9790 |
| 48 | occipital pole | 0.4889 | 0.4879 | 0.5800 | 0.0049 | 0.8329 | 0.0237 |

*Abbreviations: Pars Triangularis (PT), Pars Opercularis (PO), Anterior Division (AD), Posterior Division (PD), Superior Division (SD), Inferior Division (ID), Temporooccipital Part (TP)

Table S2. Quantitative assessment of the phantom data presented in Figure S6. The dice coefficient (DC) was computed in ROI I (yellow rectangle, Figure S6) and ROI II (green rectangle, Figure S6), with the former more affected by susceptibility because of the presence of a large air bubble. The DC values were increased following distortion correction in both ROIs, regardless of segmentation factor, particularly following the reversed-PE correction. Higher segmentation led to higher DC values.

| **Distortion Correction** | **Non-Segmented Equivalent** | | **Two-fold Segmented Equivalent** | |
| --- | --- | --- | --- | --- |
|  | **ROI I** | **ROI II** | **ROI I** | **ROI II** |
| **No correction** | 0.874 | 0.988 | 0.933 | 0.992 |
| **B0 filed mapping** | 0.967 | 0.995 | 0.982 | 0.996 |
| **Reversed-PE** | 0.979 | 0.998 | 0.984 | 0.998 |

**References**

1. Schallmo, M.P., et al., *Assessing methods for geometric distortion compensation in 7 T gradient echo functional MRI data.* Human Brain Mapping, 2021. **42**(13): p. 4205-4223.
